# High force catch bond mechanism of bacterial adhesion in the human gut

**DOI:** 10.1038/s41467-020-18063-x

**Published:** 2020-08-28

**Authors:** Zhaowei Liu, Haipei Liu, Andrés M. Vera, Rafael C. Bernardi, Philip Tinnefeld, Michael A. Nash

**Affiliations:** 1 Institute of Physical Chemistry, Department of Chemistry, University of Basel, 4058 Basel, Switzerland; 2 Department of Biosystems Science and Engineering, ETH Zurich, 4058 Basel, Switzerland; 3 Faculty of Chemistry and Center for NanoScience, Ludwig-Maximilians-Universität München, Munich, Germany; 4 NIH Center for Macromolecular Modeling and Bioinformatics, Theoretical and Computational Biophysics Group, Beckman Institute for Advanced Science and Technology, University of Illinois at Urbana-Champaign, 61801 Urbana, IL USA; 5 Department of Physics, Auburn University, 36849 Auburn, AL USA

**Keywords:** Bacterial structural biology, Mechanical properties, Applications of AFM

## Abstract

Bacterial colonization of the human intestine requires firm adhesion of bacteria to insoluble substrates under hydrodynamic flow. Here we report the molecular mechanism behind an ultrastable protein complex responsible for resisting shear forces and adhering bacteria to cellulose fibers in the human gut. Using single-molecule force spectroscopy (SMFS), single-molecule FRET (smFRET), and molecular dynamics (MD) simulations, we resolve two binding modes and three unbinding reaction pathways of a mechanically ultrastable *R. champanellensis* (*Rc*) Dockerin:Cohesin (Doc:Coh) complex. The complex assembles in two discrete binding modes with significantly different mechanical properties, with one breaking at ~500 pN and the other at ~200 pN at loading rates from 1-100 nN s^−1^. A neighboring X-module domain allosterically regulates the binding interaction and inhibits one of the low-force pathways at high loading rates, giving rise to a catch bonding mechanism that manifests under force ramp protocols. Multi-state Monte Carlo simulations show strong agreement with experimental results, validating the proposed kinetic scheme. These results explain mechanistically how gut microbes regulate cell adhesion strength at high shear stress through intricate molecular mechanisms including dual-binding modes, mechanical allostery and catch bonds.

## Abstract (web summary)

Understanding bacterial adhesion is important in a number of different areas of study. Here using a range of simulations and experimental methods, the authors, report on the molecular mechanism behind the binding of bacteria to cellulose fibers at high shear force in the human gut.

## Introduction

When cells adhere to surfaces under flow, adhesion bonds at the cell–surface interface experience mechanical tension and resist hydrodynamic drag forces. Because of this mechanical selection pressure, adhesion proteins have evolved molecular mechanisms to deal with tension in different ways. Most bonds not involved in force transduction in vivo have lifetimes that decay exponentially with applied force, a behavior well described by the classical Bell–Evans (BE) slip bond model^1–3^. Less intuitive are catch bonds^4–7^, which are receptor–ligand interactions that serve as band pass filters for force perturbations, becoming stronger with applied force and weakening when force is released. When probed in constant force mode, the lifetime of a catch bond rises as the force setpoint is increased. When probed in force ramp mode or constant speed mode, catch bonds typically give rise to bimodal rupture force distributions^8,9^. Different kinetic state models and network topologies can be used to describe catch bonds^10^. For example, mechanical allostery models such as the one-state two-pathway model^11^, or two independent sites model^8,12^ have been applied to mathematically describe catch bond behavior^6,8,12,13^.

**Fig. 1:**
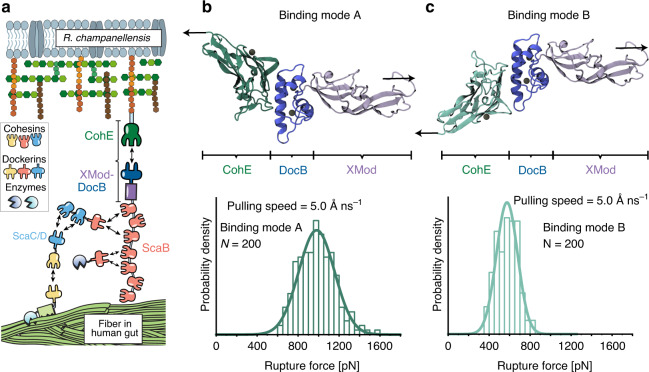
*Rc* XMod-Doc:Coh complex, dual-binding modes, and molecular dynamics simulation of complex dissociation. **a** The *Rc*-cellulosomal network is assembled through interactions between Doc and Coh domains. Cellulose-binding domains, digestive enzymes, and structural scaffold proteins (Sca) self-assemble into a cellulosome complex, which binds and digests cellulose fibers in the human gut. XMod-DocB and CohE form a mechanically stable protein complex that anchors the cellulosomal network to the cell surface. **b**, **c** Structural models showing the XMod-Doc:Coh complex in the two hypothesized-binding modes. Green: Coh, blue: Doc, purple: XMod. Calcium ions are shown as black spheres. In both binding modes, the rupture forces observed for the five most stable models were measured by performing 200 steered molecular dynamics (SMD) replicas and plotting as histograms. The pulling directions are marked by black arrows. Rupture force histograms were fitted with Gaussian distributions. The most probable rupture force was 981 pN in binding mode A (panel **b**) and 575 pN in binding mode B (panel **c**) at a pulling speed of 5.0 Å ns^−1^.

The *R. champanellensis* (*Rc*) cellulosome^14,15^ is a bacterial protein complex found in the human gut that adheres to and digests plant fiber^16^. The large supramolecular complex is held together by dockerin:cohesin (Doc:Coh) interactions^17^, which comprise a family of homologous high-affinity receptor–ligand pairs. A proposed topology of the *Rc* cellulosome is shown in Fig. 1a^14,15^. We focus here on the mechanical stability of the complex formed between DocB located at the C-terminus of Scaffoldin B, and CohE which is covalently attached to the peptidoglycan cell wall. This complex anchors the base of the cellulosome network to the cell surface, and is required in vivo to maintain cell adhesion under hydrodynamic flow and applied shear stress. Single-molecule interactions between fiber substrates and cellulose-binding modules (CBMs) have been reported to rupture at moderate forces (~50 pN)^18,19^. However, due to the multivalency of interactions the mechanical requirements on the anchoring complex are more stringent. At the same time, the complex must be able to release cellulosome complexes to facilitate dynamic niche exploration and cellulosome shedding in response to new substrates. These seemingly mutually exclusive requirements of dynamics and strong adhesion motivated us to understand the molecular mechanism of the anchoring complex in more detail.

*Rc*-DocB belongs to the type III dockerin family and is the only *Rc* dockerin with an adjacent X-module domain (XMod; Fig. 1a, purple)^14,15^. XMod was previously shown in a related system from *Ruminococcus flavefaciens* (*Rf*) to stabilize Doc and increase the mechanical stability of the XMod-Doc:Coh complex^20,21^. Furthermore, the binding helices of *Rc-*DocB are highly symmetric (Supplementary Fig. 1, helices 1 and 3), a feature observed in type I and II Doc:Coh complexes that exhibit dual-binding modes^22–27^. Dual-binding modes arise in certain Doc:Coh complexes where the complex populates two distinct binding conformations involving different sets of binding residues on Doc recognizing the same residues on Coh. In these systems, due to structural and sequence symmetry, Doc can be rotated 180° with respect to Coh to form an alternative bound conformation. However, dual binding mode behavior has not been previously reported in type III Docs, such as the *Rc* XMod-Doc:Coh complex reported here.

Observing dual binding modes experimentally is extremely challenging using conventional bulk experiments because the two binding modes have nearly identical equilibrium binding affinity. Instead, we take a single-molecule approach which is uniquely suited for studying discrete heterogeneous systems. Single-molecule force spectroscopy with the atomic force microscope (AFM-SMFS) is able to explore a large force range up to several nN and has been used to characterize protein-folding pathways^28–30^ and receptor–ligand interactions^31–34^. Single-molecule FRET (smFRET) is capable of measuring distances at the molecular scale^35^, and has been used to study protein dynamics^36,37^ and to characterize structures of receptor–ligand complexes^38,39^. At the computational level, these experimental single-molecule approaches can be elaborated upon by employing molecular dynamics (MD) simulations^40^. When combined, these experimental and computational approaches can provide mechanistic insights into the dynamics of receptor–ligand complexes^32,41^.

Here we use AFM-SMFS, smFRET, and MD simulations to study putative dual-binding modes and catch bond behavior of the *Rc* XMod-Doc:Coh complex. We develop a state map with experimentally measured transition rates to fully describe the system, and perform kinetic Monte Carlo simulations that recapitulate the experimental data. What emerges from this three-state kinetic scheme is a picture of a unique adhesion bond that resembles a catch bond when probed under force ramp conditions, but maintains slip bonding under constant force.

## Results

### XMod-Doc:Coh homology model and expression cassettes

Since no structural information was available for the *Rc* XMod-Doc:Coh complex, we created homology models of each protein domain using Modeler 9.22^42^. The structure of the *Rc* XMod-Doc domain was modeled based on the available structure of *Rf* CttA XMod-Doc (PDB 4IU3)^43^, which shares 20% sequence identity (35% similarity) with the *Rc* domain. The structure of the *Rc* Coh domain was modeled based on two different available structures from *Rf*, namely CohE (PDB 4IU3) with 15% sequence identity (28% similarity), and CohG (PDB 4WKZ)^44^ with 18% sequence identity (34% similarity). Full amino acid sequences are given in the Supplementary Note 1. The selected templates share very high structural similarity with previously reported Coh and Doc domains. The 10 models with highest score from Modeler were selected for each domain/template pair, resulting in 10 models for the *Rc* XMod-Doc domain, and 20 models for the *Rc* Coh domain. Employing VMD^45^, we assembled 200 models of the *Rc* XMod-Doc:Coh complex in each of the two binding modes (Fig. 1b, c), building all possible combinations between XMod-Doc and Coh models. For binding mode A, the structure of the *Rf* XMod-Doc:Coh complex (PDB 4IU3) was employed to guide the *Rc* Coh:Doc interface alignment. To create a model for the hypothesized alternative binding mode B, Doc helix 1 from the homology model structure was used as a guide for the superposition of Doc helix 3. This alignment resulted in the XMod-Doc rotating 180° with respect to Coh. The models show that Doc binds Coh via the two Ca^2+^-binding loops and two binding helices (helices 1 and 3, see Supplementary Fig. 1), forming a binding interface consisting of a hydrophobic center surrounded by hydrophilic amino acids, as shown in Supplementary Fig. 2. This duplicated F-hand motif is consistent with those of other Doc domains which have been shown to exhibit dual binding modes^22,24–26^.

We cloned polyproteins containing several modules for AFM-SMFS and purified them from *E. coli*. A ddFLN4 and an elastin-like polypeptide (ELP) were used as an unfolding fingerprint domain^46^ and flexible linker^47–50^, respectively. The Coh construct (N- to C-terminus) was Coh-ddFLN4-ELP-HIS-ybbr. The XMod-Doc construct (N- to C-terminus) was ybbr-ELP-ddFLN4-XMod-Doc-HIS. The ybbR tag facilitated site-specific and covalent linkage to the coverglass or cantilever tip^51^. The loading geometry with Coh pulled from its C-terminus and XMod-Doc pulled from its N-terminus precisely mimicked that experienced by the complex in vivo. Analysis of the equilibrium binding affinity of WT XMod-Doc:Coh using isothermal titration calorimetry (ITC) revealed *K*_D_ = 1.0 ± 0.3 nM and a binding stoichiometry of 1:1. SDS–PAGE and mass spectrometry analysis indicated a molecular weight of 44 kDa for Coh construct and 55 kDa for XMod-Doc construct.

### Steered molecular dynamics (SMD) simulations reveal a weak and a strong binding mode

To examine the stability of *Rc* XMod-Doc:Coh under mechanical load we carried out SMD simulations^3^ employing NAMD^52,53^ and its QwikMD^54^ interface. First, to test the stability of the 200 models of the complex in each binding mode, we performed equilibrium MD simulations for a combined simulation time of 2.0 μs (10 ns per model), followed by a combined 8.0 μs (40 ns per model) of SMD simulations at constant pulling velocity. These SMD simulations served as a metric to eliminate unsuitable structural models. We expected that good structural models should be stable under mechanical load, therefore, for each binding mode, we selected the five strongest complexes out of the 200 models. In fact, some of the 400 complexes were found not to be stable already after the equilibrium MD, and due to the low sequence identity of the templates, most of the models were not stable under mechanical load. A visual observation in VMD showed that many of these models had only partial contact between Coh and Doc following equilibrium MD. From the five strongest models for each binding mode, we performed 200 production SMD simulation replicas, using a similar protocol as previously described^32,41^. The simulations reveal that the dissociation of XMod-Doc:Coh occurs at clearly distinct forces for the two different binding modes, with mode A dissociating at ~981 pN, and mode B at ~575 pN, both at a 5.0 Å ns^−1^ pulling speed (Fig. 1b, c).

### Wild type XMod-Doc:Coh unbinds along three distinct pathways

**Fig. 2:**
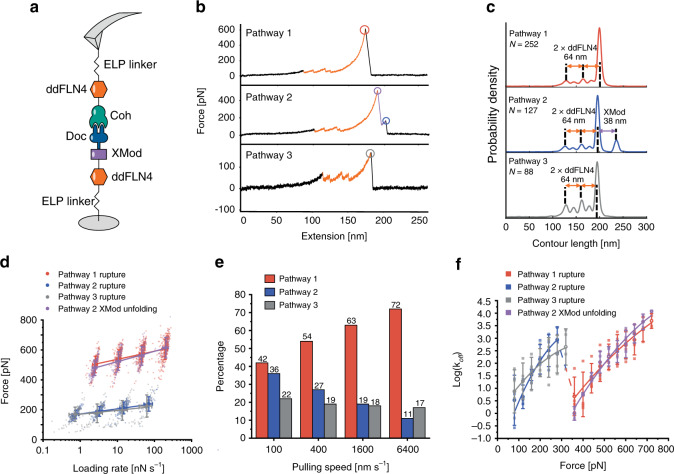
XMod-Doc:Coh unbinds along three pathways under mechanical load. **a** Experimental configuration with Coh-ddFLN4-ELP immobilized on the AFM tip and ELP-ddFLN4-XMod-Doc immobilized on the surface. Immobilization was site-specific and covalent through a terminal ybbR tag on the ELP. **b** Three different classes of force curves were repeatedly observed, corresponding to different pathways. In pathway 1 (P1), the complex ruptured at high force (500–600 pN, red circle) with the XMod remaining folded. In pathway 2 (P2), XMod unfolded (purple circle) followed by a low force rupture of the complex (blue circle). In pathway 3 (P3), the complex ruptured at low force rupture (gray circle) with the XMod remaining folded. Unfolding of the two ddFLN4 fingerprint domains (orange) was used to identify single-molecule traces. **c** Combined contour length histograms for each unbinding pathway. Force-extension traces were transformed using a freely rotating chain elasticity model and aligned using cross-correlation analysis. Histograms show contour length increments resulting from unfolding of 2× ddFLN4 and XMod. **d** Rupture force vs. loading rate plot showing final XMod-Doc:Coh complex rupture events obtained from the three pathways, as well as XMod unfolding events observed in P2. Error bars represent the standard deviation of rupture forces and loading rate (*n* = 74-317). Lines show linear Bell–Evans fits of the most probable rupture/unfolding force vs. logarithm of loading rate to obtain Δ*x*^‡^ and *k*_0_ (Eq. (2)). Fitted Δ*x*^‡^ and *k*_0_ values are listed in Table 1. **e** Percentages of the three pathways in all rupture events at different pulling speeds. **f** Kinetic off-rate (*k*_off_) vs. force for complex rupture and XMod unfolding events. Force-dependent off-rates calculated using the histogram transformation method (Eq. (3)) were plotted against force (shown in boxes). The average off-rates of four different pulling speeds were shown in open circles and fitted to the analytical expression (Eq. (5)) with *υ* = 0.5. Error bars represent the standard deviation of off-rate (*n* = 2–4). The fitted Δ*x*^‡^, Δ*G*^‡^, and *k*_0_ values are listed in Table 1.

We performed AFM-SMFS with Coh covalently attached to the cantilever tip through its C-terminal ybbR tag and XMod-Doc covalently attached to the surface (Fig. 2a) through its N-terminal ybbR tag. The XMod-Doc:Coh complex was formed by approaching the AFM tip to the surface and dwelling for 200 ms. After XMod-Doc:Coh complex formation, the cantilever base was retracted at constant speed and a force-extension curve was recorded. This procedure was repeated thousands of times typically over a 12 h period to generate large datasets of force vs. extension curves. The recorded force curves were transformed into force vs. contour length space using a freely rotating chain (FRC) elasticity model (Eq. (1)). We searched for the contour length pattern of ddFLN4, which contained ~32 nm of total contour length that resulted from a two-step unfolding pattern. Since one ddFLN4 molecule was contained in the surface-linked protein, and another one in the cantilever-linked protein, we only analyzed curves which contained in total two ddFLN4 unfolding fingerprints, thereby eliminating spurious signals.

We repeatedly observed three distinct unbinding pathways of the complex, as shown in Fig. 2b. We refer to these as pathway 1 (P1), pathway 2 (P2), and pathway 3 (P3). We used cross-correlation analysis^55,56^ to assemble superposition contour length histograms for each pathway (Fig. 2c). These histograms all showed the distinct unfolding pattern of two ddFLN4 fingerprint domains, adding in total 64 nm of contour length to the system. P2 showed an additional 38 nm length increment which matched the expected value for XMod unfolding (116 XMod amino acids × 0.365 nm/amino acid − 5.3 nm folded length = 37 nm) (Fig. 2c, middle). The contour length histograms were broadened by occasional unassigned unfolding events that were observed in all three pathways, which we attributed to partial unfolding of Coh or Doc. A representative sampling of these unassigned unfolding events are presented in Supplementary Fig. 3.

Approximately 80% of curves were assigned to P1 or P2. In P1, unfolding of two ddFLN4 domains in series was followed by dissociation of XMod-Doc:Coh at high forces of ~500 pN (Fig. 2b, top). In P2, XMod unfolded at high forces, followed by the Doc–Coh complex rupture at low forces of ~200 pN (Fig. 2b, middle). This indicated that XMod unfolding significantly destabilized the interaction between Doc and Coh in P2, giving rise to a shielded complex rupture event. P2 was reproduced in SMD simulations by deleting the XMod from the Doc:Coh complex in binding mode A, which showed a decrease in rupture forces (Supplementary Fig. 4). The remaining 20% of curves were classified as P3, where XMod-Doc:Coh ruptured at low force (~200 pN, Fig. 2b, bottom) and no XMod unfolding was observed. Based on these classifications, we hypothesized that P1 and P2 resulted from complexes with high mechanical stability, which were able to resist external forces as high as ~500 pN prior to high force complex rupture or XMod unfolding. In the cases where XMod unfolded, the Doc–Coh-binding interaction became destabilized and ruptured at low force. P3 meanwhile represented a weaker Doc–Coh complex that ruptured at lower force (~200 pN) even without XMod unfolding. The existence of complexes with different mechanical stabilities was consistent with SMD simulation results (Fig. 1c), which showed that the dual-binding modes rupture at distinct forces.

### Allosteric regulation by XMod gives rise to catch bonding

AFM measurements on WT XMod-Doc:Coh were carried out at pulling speeds of 100, 400, 1600, and 6400 nm s^−1^, which allowed us to investigate the loading rate dependency of complex rupture and XMod unfolding in the various pathways (Fig. 2d, Supplementary Fig. 5 and Supplementary Note 2). We used the BE model (Eq. (2))^1,2^ to analyze the experimental force-loading rate data and obtain the intrinsic off rate (*k*_0_) and the distance to the transition state along the reaction coordinate (Δ*x*^‡^) for the complex rupture events in each pathway, as well as for XMod unfolding along P2 (Table 1).

**Table 1:** Kinetic parameters extracted from AFM-SMFS.

Pathway	Event	*k*_0_ [s^−1^] (BE)	Δ*x*^‡^ [nm] (BE)	Δ*G*^‡^ [*k*_B_*T*] (DHS)		*k*_0_ [s^−1^] (DHS)		Δ*x*^‡^ [nm] (DHS)	
				υ = 0.5	υ = 2/3	υ = 0.5	υ = 2/3	υ = 0.5	υ = 2/3
1	High force rupture (XMod intact)	4.70 × 10^−8^	0.178	22.8	20.0	9.39 × 10^−5^	1.57 × 10^−4^	0.146	0.132
2	Unfolding of XMod (measured)	9.94 × 10^−6^	0.139	27.6	24.0	5.74 × 10^−7^	4.79 × 10^−6^	0.209	0.168
2	Unfolding of XMod (corrected)	4.53 × 10^−5^	0.116	30.1	26.1	2.66 × 10^−6^	5.42 × 10^−6^	0.173	0.157
2	Low force rupture after XMod unfold	7.43 × 10^−4^	0.277	13.9	12.5	0.0152	0.101	0.254	0.171
3	Low force rupture (XMod intact)	2.79 × 10^−5^	0.366	8.46	7.45	0.831	1.08	0.131	0.113

As shown in Fig. 2e, the percentage of curves that were classified as P3 was independent of the pulling speed, maintaining a value of 17–22% across the range of speeds tested. This observation was consistent with the hypothesis that P3 belonged to a different binding mode than P1 and P2. Interestingly, the ratio between P1 and P2 was dependent on the pulling speed. The likelihood of P1 increased with increasing pulling speed from 100 to 6400 nm s^−1^, while the likelihood of observing P2 decreased. This means that the complex preferentially populated the pathway with higher rupture force (P1) when pulled at higher loading rates. This switch from low rupture force P2 to high rupture force P1 at increasing loading rates is not to be confused with standard scaling based on BE theory, which also predicts higher rupture forces at higher loading rates. This behavior, in contrast, represented a discrete non-linear switching from P2 to P1 with much higher rupture forces. Although P1 and P2 rupture events each individually scale as classical slip bonds as a function of the loading rate, the pathway switching behavior precisely mimics that of a catch bond^4,6,12,57,58^ probed under force ramp conditions. In contrast to other reported catch bonds in the literature which occur at low force (<50 pN), XMod-Doc:Coh is activated at much higher forces (>300 pN).

The explanation for this apparent catch bond behavior under force ramp conditions is evident when looking at the loading rate dependency of XMod unfolding. The loading rate dependency of XMod unfolding is steeper than that of the complex rupture in P1 (Fig. 2d and Table 1). Therefore, at high loading rates, far fewer complexes reach sufficiently high forces to unfold XMod prior to complex rupture, thus prohibiting the system from entering P2. This behavior is unique to this particular XMod-Doc:Coh system and was not observed in other Doc:Coh systems reported thus far^20,41^.

We note that the experimentally observed values for XMod unfolding are slightly biased by the maximal stability of the receptor–ligand complex^59^. This ceiling effect is magnified at high loading rates (>100 nN/s) because the XMod unfolding force increases and exceeds the maximal force that the complex can withstand. We corrected the XMod unfolding force distribution to take this biasing effect into account^59^, as shown in Supplementary Fig. 6. Using the BE model, we obtained the kinetic parameters of XMod unfolding after bias correction (Table 1). This analysis confirmed what was observed in the rupture force vs. loading rate scatter plots, namely that XMod has a steeper loading rate dependency (lower Δ*x*^‡^) than the high force rupture event in P1, and that these scaling differences give rise to catch bonding in force ramp/constant speed mode.

The rupture forces from three pathways as well as the XMod unfolding forces obtained at different pulling speeds were plotted as histograms (Supplementary Figs. 6 and 7), which were transformed into force-dependent off rate *k*_off_(*F*) using Eq.(3) and plotted against force. The force-dependent off-rates were fitted using Dudko–Hummer–Szabo (DHS) model (Eq. (5))^60,61^ to extract the intrinsic barrier crossing rate (*k*_0_), barrier height (Δ*G*^‡^) and distance to the transition state along the reaction coordinate (Δ*x*^‡^) of the various barrier-crossing events, as shown in Fig. 2f, Supplementary Fig. 8 and Table 1. The off rate vs. force plot was fitted using two different *υ* values in Eq. (5): *υ* = 0.5 (Fig. 2f, assuming a cusp-like energy barrier) and *υ* = 2/3 (Supplementary Fig. 8, assuming a linear-cubic energy barrier). Both *υ* values generated good fits and extracted similar *k*_0_, Δ*G*^‡^ and Δ*x*^‡^ values (Table 1), meaning that the model is applicable to the experimental data. In addition to the transformation shown in Fig. 2f, we used Eq. (6) to calculate the force-dependent off-rate based on combining the histograms from P1 and P2 rupture events (Supplementary Fig. 9)^62,63^. The rationale for combining the analysis of P1 and P2 rupture events was motivated by the evidence for dual-binding modes (see below). The force-dependent off-rate calculated from both separated and combined P1/P2 histograms (Fig. 2f and Supplementary Figs. 8 and 9) showed a crossover regime around 300 pN where the off rate decreased with increasing force, consistent with previously reported multi-pathway systems leading to catch bond behavior^62,63^. We note that comparison of the fitted energy landscape parameters between these two models in Table 1 is complicated by the fact that the BE model assumes Δ*x*^‡^ is constant and independent of the force, while the DHS model assumes both Δ*x*^‡^ and Δ*G*^‡^ to be force-dependent. In practice we used DHS model to generate off-rates for each pathway at constant force with data derived from constant speed experiments, while BE fitting was used for extracting the loading rate dependency of rupture events.

### AFM-SMFS evidence of dual-binding modes

**Fig. 3:**
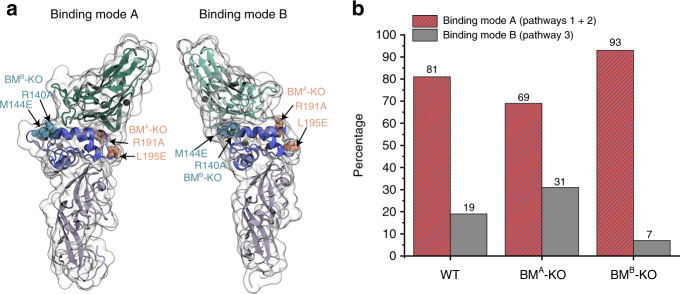
SMFS measurements of XMod-Doc-binding mode mutants. **a** Arginine 191 and leucine 195 on Doc were mutated to alanine and glutamic acid, respectively, to disrupt their interaction with Coh in binding mode A. Arginine 140 and methionine 144 were mutated to alanine and glutamic acid, respectively, to disrupt their interaction with Coh in binding mode B. **b** Percentages of the two binding modes measured with WT XMod-Doc, BM^A^-KO mutant, and BM^B^-KO mutant at 400 nm/s pulling speed. The BM^A^-KO mutant preferentially populates binding mode B while the BM^B^-KO mutant preferentially populates binding mode A.

We hypothesized that P1 and P2 arose from one binding mode, while P3 arose from an alternative binding mode with lower mechanical stability. To test this, we sought to knock out specific binding modes by mutagenesis (Fig. 3a and Supplementary Fig. 1). Using the structural models, we identified key Doc residues likely to be involved in each respective binding mode (Supplementary Fig. 1), and designed mutations to disrupt electrostatics and hydrogen bonding. The mutant designed to knock out binding mode A contained R191A and L195E mutations, and is referred to as BM^A^-KO. The mutant designed to knock out binding mode B contained R140A and M144E mutations and is referred to as BM^B^-KO. Interactions between BM^A^-KO or BM^B^-KO and Coh were then measured using AFM-SMFS at 400 nm/s. For WT XMod-Doc:Coh, the percentage of P3 curves was typically ~20%. As shown in Fig. 3b and Supplementary Fig. 10, BM^A^-KO resulted in a P3 curve percentage that increased to 31%. We attributed this increase in P3 probability to the destabilization of binding mode A, and slight preferential formation of binding mode B as compared to WT. This result indicated that binding mode B was likely associated with the low force pathway P3. However, the mutations were not able to completely knock out binding mode A.

BM^B^-KO was more effective at knocking out binding activity, and decreased the percentage of P3 curves from 19% for WT down to 7% with a corresponding increase in P1 and P2 percentages. Despite the introduction of destabilizing mutations at the binding interface in BM^B^-KO, we nonetheless obtained a system with higher stability and predominantly high force rupture pathways, a result that may seem counterintuitive but is explained by the presence of a weak binding mode B being knocked out or inhibited by the mutations. Based on these measurements with the binding mode knock-out mutants, we concluded that P1 and P2 are attributable to binding mode A, which is the strong binding mode, while P3 corresponds to binding mode B, which is the weak binding mode. In contrast to other Doc:Coh systems exhibiting dual-binding mode^24,25^, the two binding modes of *Rc* XMod-Doc:Coh complex have significantly different mechanical stabilities with one rupturing at ~200 pN and the other able to withstand forces of ~500–600 pN.

This conclusion was further supported by a statistical analysis involving a biasing effect of an additional fingerprint domain^59^ (see Supplementary Note 3 and Supplementary Fig. 11). We introduced an additional fingerprint domain (I27) whose unfolding force sits in between the P1 and P3 rupture events. If the multi-pathway dissociation behavior that we observed resulted from multiple unbinding reaction pathways originating from a single bound state, we would expect that the likelihood of observing an I27 unfolding event would be decorrelated from the pathway classification of the curve. We did not observe this, and instead the vast majority of curves that showed I27 unfolding terminated in a high force rupture event (P1) or XMod unfolding followed by low force rupture (P2). This indicated that complexes that ruptured in a low force rupture event (P3) were not sufficiently strong to unfold I27, consistent with P3 emerging from a discrete binding mode that was weaker than the P1 or P2 complex, further substantiating the dual binding modes.

### smFRET evidence of dual-binding modes

**Fig. 4:**
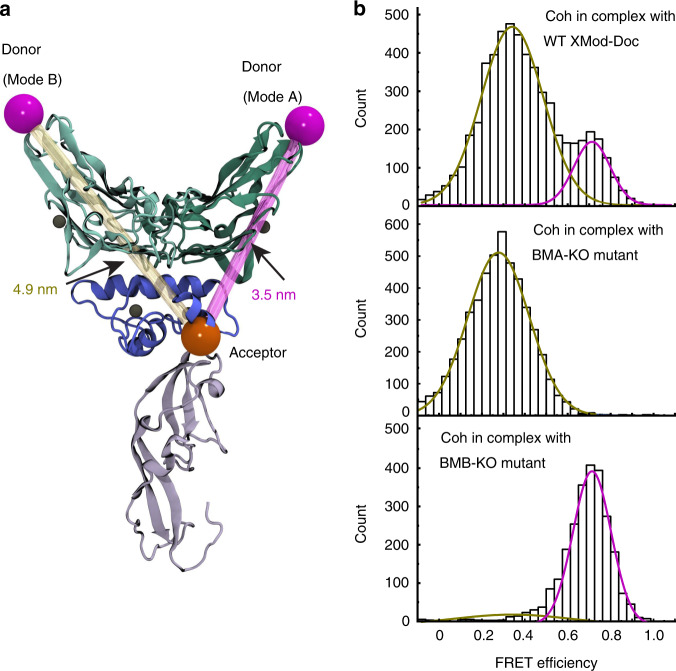
Dual binding modes are observed by smFRET. **a** The C-terminus of Coh (position 154) was labeled with the FRET donor Cy3b (shown in magenta) while the C terminus of the XMod-Doc (position 199) was labeled with the FRET acceptor AF647 (shown in orange). The donor–acceptor distance was smaller in binding mode A (3.5 nm) than the binding mode B (4.9 nm), giving rise to a higher FRET efficiency when the complex formed in binding mode A. **b** FRET efficiency histograms measured using Cy3b-labeled WT XMod-Doc (top), BM^A^-KO (middle), or BM^B^-KO (bottom) complexed with AF647-labeled Coh. Efficiency histograms were fitted with single or double Gaussian distributions.

Based on differences in inter-residue distances in the two binding conformations, we used smFRET to observe the dual-binding modes. We introduced a point cysteine mutation at position 154 of Coh and covalently attached a FRET donor dye maleimide-Cy3b. Since XMod-Doc has native cysteines, we used amber suppression^64^ to introduce a non-canonical azide at position 199 of XMod-Doc, and covalently attached DBCO-AF647. Based on the homology models (Fig. 4a), the donor–acceptor distance is expected to be ~3.5 nm in binding mode A and ~4.9 nm in binding mode B. XMod-Doc:Coh complexes were formed by mixing labeled XMod-Doc and Coh in a 1:1 molar ratio and diluting them to ~200 pM. FRET efficiency of individual XMod-Doc:Coh complexes was measured on a confocal microscope and plotted into histograms (Fig. 4b). A bimodal distribution was clearly observed in the FRET efficiency histogram of WT XMod-Doc:Coh, with mean FRET efficiencies of 0.34 and 0.71, corresponding to binding modes B and A, respectively. In addition to labeling and analyzing WT, we introduced the FRET acceptor dye into BM^A^-KO and BM^B^-KO mutants at position 199, and again measured FRET efficiency in complex with labeled Coh using the same protocol as for WT. We found that only the low FRET efficiency peak was observed in BM^A^-KO, meaning that binding mode A corresponding to the high FRET efficiency peak was eliminated by the mutations. The FRET efficiency histogram of BM^B^-KO complexed with Coh meanwhile showed predominantly the high FRET efficiency population, consistent with binding mode B being knocked out. Compared to AFM-SMFS, the binding mode A is much less prevalent in the smFRET measurement of wild-type complex and the BM^A^-KO mutant knocks out the binding mode A much more efficiently in smFRET measurement. We attributed this difference to the acceptor dye destabilizing the complex in binding mode A but not binding mode B, which was supported by AFM-SMFS measurements between dye-labeled BM^A^-KO and unlabeled Coh (Supplementary Fig. 12).

### Kinetic model and Monte Carlo simulations

**Fig. 5:**
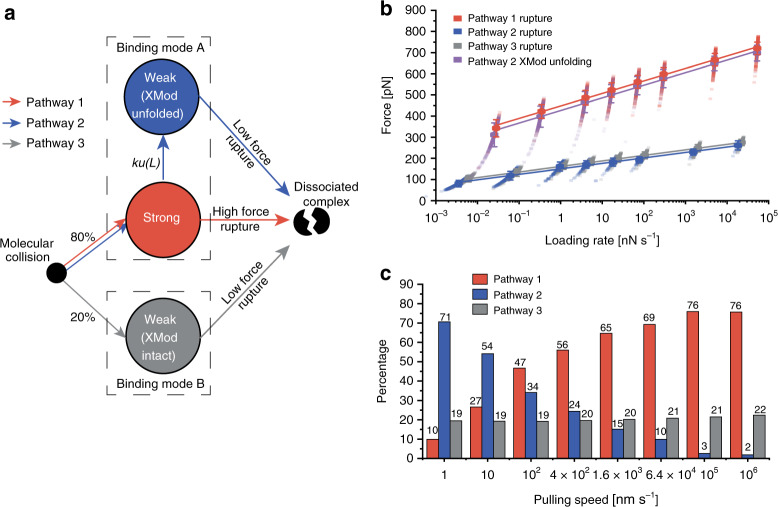
Multi-state kinetic model and Monte-Carlo simulation of mechanical rupture of XMod-Doc:Coh. **a** The multi-state kinetic model postulates that upon molecular collision, the complex has ~80% probability of forming the strong binding mode A (pathways 1 and 2) and 20% probability of forming the weaker binding mode B (pathway 3). Once the binding mode is set, there is no interconversion between the modes. In the strong binding mode, the complex can rupture at high force (pathway 1) with XMod remaining folded, or XMod can unfold prior to complex rupture according to a loading rate-dependent unfolding rate *k*_u_(*L*) (pathway 2). In pathway 2, XMod unfolding destabilizes the complex, resulting in low force complex rupture. At increased loading rates, the XMod unfolding rate decreases so that pathway 2 is deactivated and the complex has a higher probability of unbinding along pathway 1 (high force rupture). In pathway 3, the complex ruptures at low force without reaching sufficiently high force to unfold XMod. **b**, **c** Monte-Carlo simulation results. Force vs. extension curves at constant pulling speed were simulated to obtain the loading rate dependency of complex rupture and XMod unfolding events **b**, as well as the percentages of the three unbinding pathways at different pulling speeds **c**. The simulation was carried out at the same pulling speeds as the experiments (100, 400, 1600, 6400 nm/s) and further extended over a range from 1 to 10^6^ nm/s. Simulated force vs. loading rate plots were fitted with the Bell–Evans model to extract *k*_0_ and Δ*x*^‡^ values (Supplementary Table 1). The error bars in **b** represent the standard deviation of rupture forces (*n* = 192–693).

Combining the experimental results and MD simulations led us to propose a kinetic scheme for the unbinding mechanism of the *Rc* XMod-Doc:Coh complex that accounts for dual-binding modes as well as the catch bond behavior observed under force ramp conditions (Fig. 5a). Our model postulates that there are two non-interconvertible bound states with different mechanical stabilities (binding modes A and B). Upon binding, the complex has an 80% probability of forming the more stable binding mode A, and a 20% probability of forming binding mode B. If the complex forms in binding mode B, the only escape pathway under load is P3 terminating in a low force rupture (~200 pN). When bound in binding mode A, the complex either ruptures at high force (P1), or enters a weakened state due to the unfolding of XMod (P2). The rate of entering the weaker state (P2) from the stronger state (P1) decreases as the loading rate increases because of the steeper loading rate dependency of XMod unfolding. This results in an increased proportion of P1 high rupture force curves when the complex is probed using force ramp conditions at high loading rates (>100 nN s^−1^), which is precisely what is observed in classical catch bonds.

We used kinetic rates obtained from AFM-SMFS combined with our proposed state model to simulate the system in a constant pulling speed scenario, identical to the experiments. We used worm-like chain elasticity theory and Monte-Carlo^65^ to simulate force-extension curves of the XMod-Doc:Coh stretching, XMod unfolding, and complex rupture (see section “Methods”—Monte Carlo simulation). The loading rate dependency of the complex rupture force and XMod unfolding forces, as well as the rupture and unfolding force histograms from the simulated curves are shown in Fig. 5b, Supplementary Figs. 13, and  14. The simulations showed remarkable agreement with experiment results both in terms of the rupture forces, and other observed trends (Fig. 2, Supplementary Figs. 6 and  7). For example, our network Monte-Carlo modeling shows the same bimodality of the rupture force distributions, similar force magnitudes and similar ratios between the P1, P2, and P3 trajectories. Furthermore, the catch bond network topology that emerged in force ramp mode was also observed in the simulation.

The simulations further allowed us to probe a range of pulling speeds that were not accessible experimentally. We extended the range of pulling speeds in the simulations to get a clearer picture of the catch bond behavior. As shown in Fig. 5c, at high loading rates, the complex predominantly ruptures along P1 due to the strengthening of XMod. At extremely slow pulling speeds, we see in the simulation that the P1 pathway is lost and the complex only exhibits P2 and P3 low force rupture behavior. The broad agreement of the simulation with the experimental results provided strong support for the proposed kinetic scheme.

## Discussion

We discovered a mechanism by which bacteria achieve mechanically stable adhesion to crystalline fiber surfaces in the human gut, and resolved the dual binding modes of this complex using single-molecule techniques and all atom simulations. The kinetic scheme amounts to a multi-state catch bond mechanism in binding mode A (P1/P2 paths). The system starts in the high rupture force (P1, activated) state and has a certain probability of entering the low rupture force state (P2). The transition rate from P1 to P2 decreases with increasing loading rate, meaning that the low rupture force state is inhibited at high loading rates. Once the complex enters the low rupture force state, it cannot return to the high rupture force state. These features make our system distinct from the other two-state catch bond models^4,7,12,13,66^. Interestingly, the catch bond behavior emerges from a network of purely slip bonds/folds and only manifests under a force ramp or constant speed scenario. If this system is probed using constant force clamp conditions, there is no increase in lifetime as the clamping force is increased (Supplementary Fig. 15).

To further clarify the description of the system as a catch bond, we note that when considering the XMod unfolding force and the binding interface together, the maximal force that the system can withstand does not increase with increasing loading rate or clamping force (Supplementary Fig. 15). This is due to the high forces required to unfold XMod and enter the low stability P2 pathway. Nevertheless, when considering the force at which the Doc:Coh binding interface breaks (i.e. the rupture event), we find the term catch bond appropriate. The force-dependent off rates (Fig. 2f and Supplementary Figs. 8 and 9) show catch bond behavior when considering the force at the time of the rupture event independently from bond history. This mechanism emerges due to the inhibition of P1 to P2 transfer rates at high loading rates (Figs. 2e and 5c), which caused P1 rupture events to become more frequent at higher loading rates.

Based on structural modeling and analysis, we predicted that the heterogeneity of unbinding pathways was attributable to two different binding conformations, binding modes A and B. AFM-SMFS and smFRET on mutant XMod-Doc constructs designed to specifically knockout binding mode A or B supported the presence of dual binding modes with different mechanical properties. The biological significance of the two binding modes is still unclear, however we speculate that the *Rc* bacterium might switch between the low (P3) and high force catch (P1/P2) adhesion modes based on post-translational modifications or environmental factors, for example fiber substrate composition or intraluminal pH of human colon^67,68^, allowing the bacterium to respond to environment change. Our research demonstrates a complex mechanism by which bacteria regulate adhesion strength through molecular mechanisms, such as dual-binding modes, mechanical allostery, and catch bonding.

## Methods

### Reagents

All reagents were at least of analytical purity grade and were purchased from Sigma-Aldrich (St. Louis, MO, USA), Thermo Fisher Scientific (Waltham, MA, USA), GE Healthcare (Chicago, IL, USA), New England Biolabs (Ipswich, MA, USA), or ABCR GmbH (Karlsruhe, Germany).

Synthetic genes were purchased from Thermo Fisher Scientific.

Primers for cloning were purchased from Microsynth AG (Balgach, Switzerland).

All buffers were filtered through a 0.2 μm polyethersulfone membrane filter (Sarstedt, Nuembrecht, Germany) prior to use. The pH of all buffers was adjusted at room temperature.

### Homology modeling and MD simulations

To the best of our knowledge, the structure of the XMod-Doc domain from Scaffoldin B of *Rc* and its binding partner, the CohE domain from the same bacterium, has not been solved by experimental means. Using homologous structures available in the Protein Data Bank (www.pdb.org), we employed Modeler 9.22^42^ to obtain a homology model of the two *Rc* cellulosomal domains. Modeler works by setting spatial restriction to the atomic positions of the model protein, based on 3D-template structures. Using the *Rc* XMod-Doc:Coh protein sequences we performed a protein BLAST^69^, finding one satisfactory homolog template for the *Rc* XMod-Doc domain, and two for the *Rc* Coh domain. For all the templates, the sequence identity was observed to be low: 20% identity between *Rc* XMod-Doc and the *R. flavefaciens* XMod-Doc (PDB 4IU3) template; 15% identity between *Rc* Coh domain and the *Rf* Coh E (PDB 4IU3) template; 18% identity between *Rc* Coh domain and the *Rf* Coh G (PDB 4WKZ) template. Likewise, the sequence similarities were also found to be small, with 35%, 28%, and 34% respectively. Regarding the templates, both the 4WKZ^44^ and the 4IUI3^43^ structures were solved by means of x-ray crystallography, with a resolution of 1.79 and 1.97 Å, respectively.

Using Modeler, we generated 10 structural models for the *Rc* XMod-Doc domain based on its template, and 20 structural models for the *Rc* Coh domain based on its two templates (10 models for each template). Using VMD^45^, the structure of the *Rf* XMod-Doc:Coh complex was used as a guide to fit all 200 possible combinations of the *Rc* model structures into binding mode A. For the binding mode B, first an inverted *Rf* XMod-Doc:Coh binding was created by superimposing Doc helix 1 with helix 3, and helix 3 with helix 1, creating a 180° rotated Coh structure. VMD was then used again to fit all the possible 200 models of *Rc* to this inverted *R. flavefaciens* structure. Typically the best structural model could be selected by employing tools like PROCHECK^70^ and ERRAT server^71^, however, due to the low sequence identity and similarity, we adopted a strategy of using MD to thoroughly test all the homology models.

Employing QwikMD^54^, all 400 model structures were subjected to 5 ns of equilibrium MD to ensure conformational stability. Although after a visual inspection we could see that many of the structural models were not stable following MD simulation, we chose to use a more systematic metric to select the best structural models, namely, selecting, for each of the binding modes, the five most stable models under load. For that we performed 20 ns of SMD for each of the 400 model structures, pulling the complex apart. The simulations revealed that the complexes would rupture at a wide-range of forces, and the five models with highest rupture forces for each binding mode were selected as the best models.

To investigate the stability of the best structural models, we performed another set of SMD simulations using the five best models as initial structures in what we call an in silico force-spectroscopy approach^40^. Using a wide-sampling strategy, 200 SMD replicas were carried out for a total of 8 μs for each binding mode, using the five different initial structures. All SMD simulations^3^ were performed with a constant velocity protocol using 5.0 Å/ns as the pulling speed. In all simulations, SMD was employed by restraining the position of the N-terminal of XMod-Doc domain, while pulling on the C-terminus of the Coh domain.

To reproduce the scenario where the XMod had unfolded, we performed another set of SMD simulations where the XMod was removed using QwikMD. Using a wide-sampling strategy, 20 SMD replicas were carried out for a total of 800 ns. This new set of SMD was performed by restraining the position of the N-terminal of Doc domain, while pulling on the C-terminus of the Coh domain.

In our study, all MD and SMD simulations were performed employing the NAMD MD package^52,53^. The CHARMM36 force field^72^ was employed to describe all simulations, using an explicit TIP3 water model^73^. Simulations were performed at the NpT ensemble, in periodic boundary conditions. Temperature was kept at 300 K using Langevin dynamics for temperature coupling, while a Langevin piston was employed to hold pressure at 1 bar. A distance cut-off of 14.0 Å was applied to short-range, non-bonded interactions, whereas the particle-mesh Ewald (PME) method was employed for long-range electrostatic interactions. The equations of motion were integrated using a 2 fs time step for all simulations performed. All simulations were analyzed using VMD^45^ and its plugins. Surface contact areas of interacting residues/domains were studied using PyContact^74^.

### Cloning

The constructs for AFM measurements were ybbr-ELP-ddFLN4-XMod-Doc-HIS and Coh-ddFLN4-ELP-HIS-ybbr. A ddFLN4 domain was inserted into a pET28a vector containing ybbr-HIS-ELP (for XMod-Doc) or ELP-HIS-ybbr (for Coh) so that the ELP linker was located between the ddFLN4 and the ybbr tag. The XMod-Doc synthetic gene was inserted to the C terminus of ddFLN4 using Gibson assembly and the Coh synthetic gene was inserted to the N terminus of ddFLN4 using restriction digestion cloning (NdeI and BamHI sites). The sequences of the inserted genes were confirmed by Sanger sequencing (Microsynth AG). The His-tag on the XMod-Doc constructs were then moved to the C terminus of the construct.

Protein samples for ITC measurement were prepared by removing ELP and ddFLN4 domains from the AFM measurement constructs.

The Coh smFRET construct was prepared by adding an Avi-tag to the N terminus of the ITC construct and introducing E154C mutation to Coh.

The WT XMod-Doc smFRET construct was prepared by replacing the serine at position 199 with an Amber codon (TCC → TGA). The smFRET constructs of the XMod-Doc-binding mode mutants were prepared by adding the same mutations as the corresponding AFM constructs to the WT XMod-Doc smFRET construct.

### Protein expression and purification

All protein samples used for AFM and ITC as well as Coh used in smFRET were expressed in NiCo21 (DE3) cells (New England Biolabs). Cells were cultured in TB (terrific broth) medium containing 50 μg/mL kanamycin until OD600 reached ~0.6. Protein expression was induced by adding 0.5 mM IPTG to the culture, followed by incubating at 20 °C overnight. Cells were harvested and lysed using sonication. The cell lysate was pelleted and the supernatant was loaded onto a His-tap FF 5 mL column (GE Healthcare) and washed with TBS buffer supplemented with calcium (TBS-Ca, 25 mM Tris, 72 mM NaCl, 1 mM CaCl_2_, pH 7.2). Bound protein was eluted using TBS-Ca buffer containing 500 mM imidazole. Eluted protein was further purified using Superose 6 10/300 GL size-exclusion column (GE Healthcare). Protein solutions for long-term storage were concentrated using a Vivaspin 6 centrifugal filter (molecular weight cut-off 5 kDa, GE Healthcare) and stored in 35% (v/v) glycerol at −20 °C. The concentration of the protein stocks were determined to be ~40 µM using UV absorption spectrophotometry.

### Amber suppression

The Doc smFRET constructs were expressed in BL21Star (DE3) cells using amber codon suppression^64^. The pET28a vector carrying the Doc smFRET construct was co-transformed with plasmid pEVOL-pAzF (a gift from Peter Schultz, Addgene plasmid #31186) to BL21Star (DE3) competent cells. Transformed cells were grown in LB medium containing 50 μg/mL kanamycin and 25 μg/mL chloramphenicol until OD600 reached ~0.8. Cells were then pelleted, washed with M9 minimal medium and resuspended in M9 medium containing 50 μg/mL kanamycin, 25 μg/mL chloramphenicol, 0.2 mg/mL *p*-azido-l-phenylalanine (pAzF, ABCR GmbH), and 0.02% arabinose. The culture was incubated at 37 °C for 1 h and then 1 mM IPTG was added to the culture, followed by incubating at 16 °C overnight. The expressed protein was extracted and purified using the same protocol as for the AFM constructs.

### AFM sample preparation

Biolever mini AFM cantilevers (Bruker, Billerica, MA, USA) and cover glasses were cleaned by UV-ozone treatment (cantilevers) or piranha solution (cover glasses), and silanized using (3-aminopropyl)-dimethyl-ethoxysilane (APDMES, ABCR GmbH) to introduce amine groups on the surface. The silanized cantilevers and cover glasses were subsequently incubated with 10 mg/mL sulfosuccinimidyl 4-(N-maleimidomethyl)cyclohexane-1-carboxylate (sulfo-SMCC, Thermo Fisher Scientific) solution for 30 min at room temperature in order to introduce maleimide groups on the surface. After incubating with sulfo-SMCC, the cantilevers and glasses were cleaned with ultrapure water and immediately incubated with 20 mM coenzyme A (CoA) solution for 2 h at room temperature and then cleaned with ultra pure water. CoA-coated cantilevers and cover glasses were incubated with Coh-ddFLN4-ELP-ybbR and ybbR-ELP-ddFLN4-XMod-Doc fusion proteins, respectively, in the presence of ~5 μM Sfp (phosphopantetheinyl transferase) enzyme and 10 mM MgCl_2_ for 2 h at room temperature. After incubation, cantilevers and glass surfaces were intensively rinsed with TBS–Ca buffer and stored under TBS–Ca buffer before measurement.

### AFM-SMFS measurements

SMFS measurements were performed on a Force Robot AFM (JPK instruments, Berlin, Germany). Cantilever spring constants (ranging from 0.07 to 0.1 N m^−1^) were calibrated using the contact-free method. A control experiment was done showing that the contact-free calibration method gave the same result as contact-based method. The cantilever was brought into contact with the surface and withdrawn at constant speed ranging from 100 to 6400 nm s^−1^. After recording each force-extension curve, the glass surface was moved horizontally by 100 nm. In a typical measurement around 5000–10,000 force-extension curves were obtained with a single cantilever in an experimental run of 10–20 h. The majority of the data were unusable curves due to lack of interactions, multiple interactions or nonspecific adhesion of molecules to the cantilever tip. However, ~10% of the curves showed single-molecule interactions. We filtered the data by searching for the two-step unfolding patterns and the 64 nm contour length increment of two ddFLN4 fingerprint domains.

### AFM data analysis

AFM data were analyzed using a combination of Python scripts, R scripts (R foundation, utilizing packages readr and ggplot2, and user interface R Studio), and Origin 2018 (OriginLab).

Force-extension curves were transformed into contour length space using FRC model, which assumes bonds of length *b* are connected by a fixed angle *ϒ*. The force-extension curves were transformed to contour length *L* using Eq. (1)^75^:

1 $$L = \left\{ {\begin{array}{ll} {\frac{{3k_{\mathrm{B}}T}}{{xFa}}\quad\quad\ \hskip-4pt{\mathrm{for}} \, \frac{{Fb}}{{k_{\mathrm{B}}T}} {\,} < {\,} \frac{l}{p}} \\ {\frac{x}{{1 - \left( {\frac{{4Fl}}{{k_{\mathrm{B}}T}}} \right)^{ - 0.5}}}\quad {\mathrm{for}} \, \frac{b}{l} {\,} < {\,} \frac{{Fb}}{{k_{\mathrm{B}}T}} {\,} < {\,} \frac{l}{b}} \\ \hskip -10pt {\frac{x}{{1 - \left( {\frac{{2Fb}}{{k_{\mathrm{B}}T}}} \right)^{ - 1}}} \quad \hskip-2pt{\mathrm{for}} \, \frac{{Fb}}{{k_{\mathrm{B}}T}} {\,} < {\,} \frac{l}{b}} \end{array}} \right.$$

where *k*_B_ is the Boltzmann constant, *T* is the temperature, $a = b\frac{{1 + {\mathrm{cos}}\gamma }}{{\left( {1 - {\mathrm{cos}}\gamma } \right){\mathrm{cos}}\frac{\gamma }{2}}}$ is the Kuhn length and $l = b\frac{{{\mathrm{cos}}\frac{\gamma }{2}}}{{\left| {{\mathrm{ln}}\left( {{\mathrm{cos}}\gamma } \right)} \right|}}$ is the persistence length.

The force-extension curves were screened using the ~64 nm contour length increment of two ddFLN4 fingerprint domains.

The most probable rupture force of the complex and unfolding force of XMod was fitted linearly against the logarithm of loading rate (*r*_f_) to extract the zero-force off rate *k*_0_ and the distance to the energy barrier Δ*x*^‡^ using Eq. (2), as explained by the BE model^1,2^

2 $$F = \frac{{k_{\mathrm{B}}T}}{{{\Delta} x^\ddagger }}{\mathrm{ln}}\left[ {\frac{{r_{\mathrm{f}}{\Delta} x^\ddagger }}{{k_0k_{\mathrm{B}}T}}} \right]$$

where *k*_B_ is the Boltzmann constant and *T* is the temperature.

The stochastic nature of complex rupture and domain unfolding leads to biasing effect^59^, where the XMod unfolding event cannot be observed after the complex rupture. Therefore the fitted parameters of the XMod unfolding were corrected by simulation of the forced pulling process. With fixed energy barrier parameters for the complex, *k*_0_ and Δ*x*^‡^ for XMod unfolding were adjusted using least-square fitting method to yield the closest ratio of curves showing the XMod unfolding referred to the experimental observations at different pulling speeds.

The rupture force of the complex and unfolding force of XMod were plotted into histograms, transformed into force-dependent off rate values and fitted using the Dudko–Hummer–Szabo model^60,61^, as explained below.

Histograms were plotted using equal bin width Δ*F* = 40 pN. For one histogram containing *N* bins, starting from *F*_0_ and ending at *F*_N_ = *F*_0_ + *N*Δ*F*. The *k*th bin can be directly transformed into the force-dependent rate constant value using Eq. (3):

3 $$k_{{\mathrm{off}}}\left( {F_{k}} \right) = \frac{{h_{k}r\left( {F_{k}} \right)}}{{\left( {\frac{{h_{k}}}{2} + \mathop {\sum }\nolimits_{i = k + 1}^N h_{i}} \right){\Delta} F}}$$

where *k*_off_(*F*_*k*_) is the off rate under the average unfolding or rupture force of the *k*th bin, *r*(*F*_*k*_) is the average loading rate of the *k*th bin, and *h*_*k*_ is the height of the *k*th bin, which is calculated using Eq. (4)

4 $$h_{k} = \frac{{C_{k}}}{{C_{{\mathrm{tot}}}{\Delta} F}}$$

where *C*_*k*_ is the number of counts in the *k*th bin and *C*_tot_ is the total number of counts in the histogram.

Based on Kramers theory, the force-dependence of *k*_off_(*F*) can be written as Eq. (5):

5 $$k_{{\mathrm{off}}}\left(F \right) = k_0\left( {1 - \frac{{\upsilon F{\Delta} x^\ddagger }}{{{\Delta} G^\ddagger }}} \right)^{\frac{1}{\upsilon } - 1}{\mathrm{e}}^{\beta {\Delta} G^\ddagger \left[ {1 - \left( {1 - \frac{{\upsilon F{\Delta} x^\ddagger }}{{{\Delta} G^\ddagger }}} \right)^{1/\upsilon }} \right]}$$

where *k*_0_ is the intrinsic off rate in the absence of force, Δ*x*^‡^ is the distance to the energy barrier, Δ*G*^‡^ is the height of the energy barrier in the absence of force, *β*^−1^ = *k*_B_*T*, and *υ* = 0.5 or 2/3, which assumes the shape of the free-energy surface is cusp or linear-cubic.

Smooth rupture force histograms (kernel density estimation) were transformed into force-dependent off rate using Eq. (6), which is the continuous form of Eq. (3)

6 $$k_{{\mathrm{off}}}\left( F \right) = \frac{{P\left( F \right)r\left( F \right)}}{{1 - \mathop {\smallint }\nolimits_0^F P\left( f \right){\mathrm{{d}}}f}}$$

where *P*(*F*) is the rupture force distribution and *r*(*F*) is the loading rate.

### smFRET sample and chamber preparation

The Coh smFRET construct was reduced by adding 5 mM tris(2-carboxyethyl)phosphine (TCEP, Sigma-Aldrich) and incubating at room temperature for 30 min. The reduced protein was mixed with 20-fold excess of maleimide-Cy3b (GE Healthcare) and incubated at room temperature for 1 h followed by incubation at 4 °C overnight. The XMod-Doc smFRET constructs incorporated with *p*-azido-phenylalanine were labeled by mixing with five-fold excess of DBCO-AF647 (Jena Bioscience, Jena, Germany) and incubating at room temperature for 1 h, followed by incubation at 4 °C overnight. The labeled Coh and XMod-Doc constructs were purified using a HiPrep 26/10 desalting column (GE Healthcare) followed by Superose 6 10/300 GL size-exclusion column (GE Healthcare).

smFRET experiments were carried out in Lab Tek chambers (Lab-Tek II chambered coverglass system, Thermo Fisher Scientific). Prior to the measurement, chambers were passivated with 1 mg/mL BSA (PAA Laboratories GmbH, Germany) for at least 1 h. BSA solution was removed only before the measurement and the chamber was washed twice with PBS and once with measurement buffer (50 mM Tris pH 8, 100 mM NaCl, 1 mM CaCl_2_, 2 mM Trolox/Trolox quinone, and 1% glucose).

### smFRET measurements

Solution smFRET experiments were performed on a PIE-based^76^ home built confocal microscope based on an Olympus IX-71 inverted microscope. Two pulsed lasers (639 nm, 80 MHz, LDH-D-C-640; 532 nm, 80 MHz, LDH-P-FA-530B, both from PicoQuant GmbH, Berlin, Germany) were altered on the nanosecond timescale by a multichannel picosecond diode laser driver (PDL 828 “Sepia II”, PicoQuant GmbH) with an oscillator module (SOM 828, PicoQuant GmbH). The lasers were coupled into a single mode fiber (P3-488PM-FC, Thorlabs GmbH, Dachau, Germany) to obtain a Gaussian beam profile. Circular polarized light was obtained by a linear polarizer (LPVISE100-A, Thorlabs GmbH) and a quarter-wave plate (AQWP05M-600, Thorlabs GmbH). The light was focused by an oil-immersion objective (UPLSAPO100XO, NA 1.40, Olympus Deutschland GmbH) onto the sample. The sample was moved by a piezo stage (P-517.3CD, Physik Instrumente (PI) GmbH & Co. KG, Karlsruhe, Germany) controlled by a E-727.3CDA piezo controller (Physik Instrumente (PI) GmbH & Co. KG). The emission was separated from the excitation beam by a dichroic beam splitter (z532/633, AHF analysentechnik AG) and focused onto a 50 μm pinhole (Thorlabs GmbH). The emission light was split by a dichroic beam splitter (640DCXR, AHF analysentechnik AG) into a green (Brightline HC582/75, AHF analysentechnik AG; RazorEdge LP 532, Laser 2000 GmbH) and red (Shortpass 750, AHF Analysentechnik AG; RazorEdge LP 647, Laser 2000 GmbH) detection channel. Emission was focused onto avalanche photodiodes (SPCM-AQRH-14-TR, Excelitas Technoligies GmbH & Co. KG) and signals were registered by a time-correlated single photon counting (TCSPC)-unit (HydraHarp400, PicoQuant GmbH). The setup was controlled by a commercial software package (SymPhoTime64, Picoquant GmbH). Excitation powers of 36 and 25 µW were used for donor and acceptor lasers (as measured in front of the entrance of the microscope).

Labeled Coh and XMod-Doc samples were mixed in a molar ratio of 1:1 at a concentration of 1 µM, incubated for 1 min, and finally diluted in the chamber to a concentration of 200 pM.

### smFRET data analysis

smFRET burst selection was performed using a sliding time window burst search algorithm, with a time window of 500 µs and a minimum of four photon per time window. A threshold for burst detection of 40 photons was used^77^. In order to sort out photobleaching and blinking events, ALEX-2CDE^78^ and ׀TDX-TAA׀ filters^79^ were used. Doubly labeled XMod-Doc:Coh complexes were further selected by keeping the stoichiometry parameter between 0.2 and 0.8. Accurate FRET efficiencies^35,80^ were calculated from fluorescence intensities as

7 $$E = \frac{{I_{{\mathrm{DA}}} - \alpha I_{{\mathrm{DD}}} - \delta I_{{\mathrm{AA}}}}}{{\gamma I_{{\mathrm{DD}}} + I_{{\mathrm{DA}}} - \alpha I_{{\mathrm{DD}}} - \delta I_{{\mathrm{AA}}}}}$$

where *I*_DA_, *I*_AA_, and *I*_DD_ are the background-corrected photon counts in the acceptor channel after donor excitation, the acceptor channel after acceptor excitation, and the donor channel after donor excitation. The *α* and *δ* correction parameters are calculated from donor only and acceptor only subpopulations and accounts for spectral cross talk and direct excitation of the donor dye. The different detection efficiencies and quantum yields of fluorophores are corrected with the *γ* correction factor^35,80^.

### ITC measurement

The titration was carried out at 25 °C using VP200-ITC instrument (MicroCal, Northampton, MA, USA). The analyte was 16.1 µM Coh (lacking ELP linker and ddFLN4 domains) and the injectant was 126 µM XMod-Doc protein (lacking ELP linker and ddFLN4 domains). Both protein samples were in TBS–Ca buffer. The titration was carried out by injecting XMod-Doc dropwise into the analyte. Each drop contained 10 µL XMod-Doc solution and there was 5 min retention time between two consecutive drops so that the system could equilibrate after injecting a drop. The power required to maintain equal temperature between the sample cell and the reference cell (filled with water) was recorded. The titration was terminated after 27 injections, when the analyte (Coh) was fully saturated by the injectant (XMod-Doc).

### Monte Carlo simulation

A Monte Carlo approach based on Kramers theory was used to validate the multi-state kinetic model. The receptor–ligand dissociation in combination with fingerprint domain unfolding was simulated in a constant pulling speed protocol. Briefly, the XMod-Doc:Coh complex was randomly assigned a binding mode to be either binding mode A (80% possibility) or binding mode B (20% possibility). The corresponding kinetic parameters (*k*_0_ and Δ*x*^‡^, see Table 1) extracted from AFM-SMFS were used for the simulation. A series of force values *F*(*t*_i_) was generated on an evenly distributed extension axis *X*(*t*_i_) using a worm-like chain (WLC) model^81^. Due to the fact that the constant pulling speed protocol is achieved by the constant speed pulling of the AFM head instead of the AFM tip, a bending correction was done by converting the molecular extension *X*(*t*_i_) to the AFM head height *H*(*t*_i_) using Eq. (8):

8 $$H\left( {t_{i}} \right) = X\left( {t_{i}} \right) + \frac{{F\left( {t_{i}} \right)}}{k}$$

where *k* is the spring constant of the AFM cantilever. Then the time series could be generated based on the pulling speed *V*:

9 $$t_{{i} + 1} = t_{i} + \frac{{H\left( {t_{{i} + 1}} \right) - H\left( {t_{i}} \right)}}{V}$$

During each time slice $\left( {{\Delta} t = t_{{i} + 1} - t_{i}} \right)$, the probability of XMod-Doc:Coh rupture or protein domain unfolding was calculated using the following equation:

10 $$P\left( F \right) = 1 - {\mathrm{e}}^{ - k_{{\mathrm{off}}}\left( F \right){\Delta} t}$$

where *k*_off_(*F*) can be drawn from Eq. (11) following the BE model:

11 $$k_{{\mathrm{off}}}\left( F \right) = k_0{\mathrm{e}}^{\beta F{\Delta} x^\ddagger }$$

where *β*^−1^ = *k*_B_*T*. The dissociation probability is compared to a random number between zero and unity. If the random number is smaller than *P*(*F*) the rupture or unfolding event occurs and the corresponding force is recorded as the rupture or unfolding force. For each pulling speed, 1000 curves were generated and a histogram was drawn for the complex rupture force as well as the XMod unfolding force (Supplementary Figs. 13 and 14). For simulation under force clamp conditions, a constant force was used and 1000 curves were generated to calculate the lifetime of the complex under each applied force (Supplementary Fig. 15).

The aforementioned Monte Carlo simulations were realized using Python code (see “Code availability” section).

## Supplementary information

Supplementary Information

## Data Availability

The source data underlying Figs. 1b, c, 2b–d, f, 4b, 5b and Supplementary Figs. 3, 4b, 5–10, 11b–d, and 12–15 are provided as a Source Data file. Plasmids used in this study are deposited at Addgene. The accession codes are listed below. Addgene #153439: pET28a-ybbr-ELP-ddFLN4-XMod-Doc-HIS (wild type) Addgene #153440: pET28a-ybbr-ELP-ddFLN4-XMod-Doc-HIS (BMA-KO) Addgene #153441: pET28a-ybbr-ELP-ddFLN4-XMod-Doc-HIS (BMB-KO) Addgene #153442: pET28a-ybbr-HIS -ELP-ddFLN4-I27-XMod-Doc (wild type) Addgene #153443: pET28a-ybbr-XMod-Doc (WT, S199AzF)-HIS Addgene #153444: pET28a-ybbr-XMod-Doc (BMA-KO, S199AzF)-HIS Addgene #153445: pET28a-ybbr-XMod-Doc (BMB-KO, S199AzF)-HIS Addgene #153446: pET28a-Coh-ddFLN4-ELP-HIS-ybbr Addgene #153447: pET28a-Avi-Coh (E154C)-HIS Source data are provided with this paper.

## Source data

Source Data

## Code availability

Code for performing the Monte Carlo simulation in Python is available at https://github.com/NashLab/Monte-Carlo. Source data are provided with this paper.

## References

[1] Evans E, Ritchie K (1997). Dynamic strength of molecular adhesion bonds. Biophys. J..

[2] Bell GI (1978). Models for the specific adhesion of cells to cells. Science.

[3] Izrailev S, Stepaniants S, Balsera M, Oono Y, Schulten K (1997). Molecular dynamics study of unbinding of the avidin–biotin complex. Biophys. J..

[4] Marshall BT (2003). Direct observation of catch bonds involving cell-adhesion molecules. Nature.

[5] Thomas WE, Trintchina E, Forero M, Vogel V, Sokurenko EV (2002). Bacterial adhesion to target cells enhanced by shear force. Cell.

[6] Thomas WE, Vogel V, Sokurenko E (2008). Biophysics of catch bonds. Annu. Rev. Biophys..

[7] Huang DL, Bax NA, Buckley CD, Weis WI, Dunn AR (2017). Vinculin forms a directionally asymmetric catch bond with F-actin. Science.

[8] Pierse CA, Dudko OK (2017). Distinguishing signatures of multipathway conformational transitions. Phys. Rev. Lett..

[9] Evans E, Leung A, Heinrich V, Zhu C (2004). Mechanical switching and coupling between two dissociation pathways in a P-selectin adhesion bond. Proc. Natl Acad. Sci. USA.

[10] Prezhdo OV, Pereverzev YV (2009). Theoretical aspects of the biological catch bond. Acc. Chem. Res..

[11] Zhu C, Lou J, McEver RP (2005). Catch bonds: physical models, structural bases, biological function and rheological relevance. Biorheology.

[12] Thomas W (2006). Catch-bond model derived from allostery explains force-activated bacterial adhesion. Biophys. J..

[13] Buckley CD (2014). Cell adhesion. The minimal cadherin-catenin complex binds to actin filaments under force. Science.

[14] Ben David Y (2015). Ruminococcal cellulosome systems from rumen to human. Environ. Microbiol..

[15] Moraïs S (2016). Enzymatic profiling of cellulosomal enzymes from the human gut bacterium, *Ruminococcus champanellensis*, reveals a fine-tuned system for cohesin-dockerin recognition. Environ. Microbiol..

[16] Artzi L, Bayer EA, Moraïs S (2017). Cellulosomes: bacterial nanomachines for dismantling plant polysaccharides. Nat. Rev. Microbiol..

[17] Smith SP, Bayer EA, Czjzek M (2017). Continually emerging mechanistic complexity of the multi-enzyme cellulosome complex. Curr. Opin. Struct. Biol..

[18] King JR, Bowers CM, Toone EJ (2015). Specific binding at the cellulose binding module–cellulose interface observed by force spectroscopy. Langmuir.

[19] Griffo A (2019). Binding forces of cellulose binding modules on cellulosic nanomaterials. Biomacromolecules.

[20] Schoeler C (2014). Ultrastable cellulosome-adhesion complex tightens under load. Nat. Commun..

[21] Schoeler C (2015). Mapping mechanical force propagation through biomolecular complexes. Nano Lett..

[22] Carvalho AL (2007). Evidence for a dual binding mode of dockerin modules to cohesins. Proc. Natl Acad. Sci. USA.

[23] Cameron K (2015). Cell-surface attachment of bacterial multienzyme complexes involves highly dynamic protein–protein anchors. J. Biol. Chem..

[24] Jobst MA (2015). Resolving dual binding conformations of cellulosome cohesin-dockerin complexes using single-molecule force spectroscopy. Elife.

[25] Nash MA, Smith SP, Fontes CM, Bayer EA (2016). Single versus dual-binding conformations in cellulosomal cohesin–dockerin complexes. Curr. Opin. Struct. Biol..

[26] Pinheiro BA (2008). The *Clostridium cellulolyticum Dockerin* displays a dual binding mode for its cohesin partner. J. Biol. Chem..

[27] Brás JLA (2016). Diverse specificity of cellulosome attachment to the bacterial cell surface. Sci. Rep..

[28] Yu H, Siewny MGW, Edwards DT, Sanders AW, Perkins TT (2017). Hidden dynamics in the unfolding of individual bacteriorhodopsin proteins. Science.

[29] Li J, Li H (2018). Mechanical unfolding pathway of the high-potential iron-sulfur protein revealed by single-molecule atomic force microscopy: toward a general unfolding mechanism for iron–sulfur proteins. J. Phys. Chem. B.

[30] Beedle AEM (2018). Forcing the reversibility of a mechanochemical reaction. Nat. Commun..

[31] Cao Y, Yoo T, Li H (2008). Single molecule force spectroscopy reveals engineered metal chelation is a general approach to enhance mechanical stability of proteins. Proc. Natl Acad. Sci. USA.

[32] Milles LF, Schulten K, Gaub HE, Bernardi RC (2018). Molecular mechanism of extreme mechanostability in a pathogen adhesin. Science.

[33] Sumbul F, Rico F (2019). Single-molecule force spectroscopy: experiments, analysis, and simulations. Methods Mol. Biol..

[34] Liu H, Schittny V, Nash MA (2019). Removal of a conserved disulfide bond does not compromise mechanical stability of a VHH antibody complex. Nano Lett..

[35] Hellenkamp B (2018). Precision and accuracy of single-molecule FRET measurements—a multi-laboratory benchmark study. Nat. Methods.

[36] Wang S, Vafabakhsh R, Borschel WF, Ha T, Nichols CG (2016). Structural dynamics of potassium-channel gating revealed by single-molecule FRET. Nat. Struct. Mol. Biol..

[37] Koh HR (2018). Correlating transcription initiation and conformational changes by a single-subunit RNA polymerase with near base-pair resolution. Mol. Cell.

[38] Borgia A (2018). Extreme disorder in an ultrahigh-affinity protein complex. Nature.

[39] Holmstrom ED, Liu Z, Nettels D, Best RB, Schuler B (2019). Disordered RNA chaperones can enhance nucleic acid folding via local charge screening. Nat. Commun..

[40] Verdorfer T (2017). Combining in vitro and in silico single molecule force spectroscopy to characterize and tune cellulosomal scaffoldin mechanics. J. Am. Chem. Soc..

[41] Bernardi RC (2019). Mechanisms of nanoNewton mechanostability in a protein complex revealed by molecular dynamics simulations and single-molecule force spectroscopy. J. Am. Chem. Soc..

[42] Webb B, Sali A (2017). Protein structure modeling with MODELLER. Methods Mol. Biol..

[43] Salama-Alber, O., Jobby, M. K. & Chitayat, S. Atypical cohesin-dockerin complex responsible for cell surface attachment of cellulosomal components BINDING FIDELITY, PROMISCUITY, AND STRUCTURAL …. *J. Biol.* **288**, 16827–16838 (2013). 10.1074/jbc.M113.466672 PMC3675615 23580648

[44] Voronov-Goldman M (2015). Standalone cohesin as a molecular shuttle in cellulosome assembly. FEBS Lett..

[45] Humphrey W, Dalke A, Schulten K (1996). VMD: visual molecular dynamics. J. Mol. Graph..

[46] Schwaiger I, Kardinal A, Schleicher M, Noegel AA, Rief M (2004). A mechanical unfolding intermediate in an actin-crosslinking protein. Nat. Struct. Mol. Biol..

[47] Ott W, Nicolaus T, Gaub HE, Nash MA (2016). Sequence-independent cloning and post-translational modification of repetitive protein polymers through sortase and sfp-mediated enzymatic ligation. Biomacromolecules.

[48] Ott W (2017). Elastin-like polypeptide linkers for single-molecule force spectroscopy. ACS Nano.

[49] Ta DT, Vanella R, Nash MA (2017). Magnetic separation of elastin-like polypeptide receptors for enrichment of cellular and molecular targets. Nano Lett..

[50] Ta DT, Vanella R, Nash MA (2018). Bioorthogonal elastin-like polypeptide scaffolds for immunoassay enhancement. ACS Appl. Mater. Interfaces.

[51] Yin J (2005). Genetically encoded short peptide tag for versatile protein labeling by Sfp phosphopantetheinyl transferase. Proc. Natl Acad. Sci. USA.

[52] Phillips JC (2020). Scalable molecular dynamics on CPU and GPU architectures with NAMD. J. Chem. Phys..

[53] Melo MCR (2018). NAMD goes quantum: an integrative suite for hybrid simulations. Nat. Methods.

[54] Ribeiro JV (2016). QwikMD—integrative molecular dynamics toolkit for novices and experts. Sci. Rep..

[55] Puchner EM, Franzen G, Gautel M, Gaub HE (2008). Comparing proteins by their unfolding pattern. Biophys. J..

[56] Jobst MA, Schoeler C, Malinowska K, Nash MA (2013). Investigating receptor-ligand systems of the cellulosome with AFM-based single-molecule force spectroscopy. J. Vis. Exp.

[57] Thomas W (2008). Catch bonds in adhesion. Annu. Rev. Biomed. Eng..

[58] Chakrabarti S, Hinczewski M, Thirumalai D (2017). Phenomenological and microscopic theories for catch bonds. J. Struct. Biol..

[59] Schoeler C, Verdorfer T, Gaub HE, Nash MA (2016). Biasing effects of receptor-ligand complexes on protein-unfolding statistics. Phys. Rev. E.

[60] Dudko OK, Hummer G, Szabo A (2006). Intrinsic rates and activation free energies from single-molecule pulling experiments. Phys. Rev. Lett..

[61] Dudko OK, Hummer G, Szabo A (2008). Theory, analysis, and interpretation of single-molecule force spectroscopy experiments. Proc. Natl Acad. Sci. USA.

[62] Utjesanovic M, Matin TR, Sigdel KP, King GM, Kosztin I (2019). Multiple stochastic pathways in forced peptide-lipid membrane detachment.. Sci. Rep..

[63] Matin TR (2020). Characterizing the locus of a peripheral membrane protein–lipid bilayer interaction underlying protein export activity in *E. coli*. Langmuir.

[64] Chin JW (2002). Addition of p-azido-l-phenylalanine to the genetic code of Escherichia coli. J. Am. Chem. Soc..

[65] Rief M, Fernandez JM, Gaub HE (1998). Elastically coupled two-level systems as a model for biopolymer extensibility. Phys. Rev. Lett..

[66] Kim J, Zhang C-Z, Zhang X, Springer TA (2010). A mechanically stabilized receptor–ligand flex-bond important in the vasculature. Nature.

[67] Yoav S (2017). How does cellulosome composition influence deconstruction of lignocellulosic substrates in *Clostridium* (*Ruminiclostridium*) *thermocellum* DSM 1313?. Biotechnol. Biofuels.

[68] Fallingborg J (1999). Intraluminal pH of the human gastrointestinal tract. Dan. Med. Bull..

[69] Altschul SF, Gish W, Miller W, Myers EW, Lipman DJ (1990). Basic local alignment search tool. J. Mol. Biol..

[70] Laskowski RA, MacArthur MW, Moss DS, Thornton JM (1993). PROCHECK: a program to check the stereochemical quality of protein structures. J. Appl. Crystallogr..

[71] MacArthur MW, Laskowski RA, Thornton JM (1994). Knowledge-based validation of protein structure coordinates derived by X-ray crystallography and NMR spectroscopy. Curr. Opin. Struct. Biol..

[72] Best RB (2012). Optimization of the additive CHARMM all-atom protein force field targeting improved sampling of the backbone ϕ, ψ and side-chain χ1 and χ2 dihedral angles. J. Chem. Theory Comput..

[73] Jorgensen WL, Chandrasekhar J, Madura JD, Impey RW, Klein ML (1983). Comparison of simple potential functions for simulating liquid water. J. Chem. Phys..

[74] Scheurer M (2018). PyContact: rapid, customizable, and visual analysis of noncovalent interactions in MD simulations. Biophys. J..

[75] Livadaru L, Netz RR, Kreuzer HJ (2003). Stretching response of discrete semiflexible polymers. Macromolecules.

[76] Müller BK, Zaychikov E, Bräuchle C, Lamb DC (2005). Pulsed interleaved excitation. Biophys. J..

[77] Nir E (2006). Shot-noise limited single-molecule FRET histograms: comparison between theory and experiments. J. Phys. Chem. B.

[78] Tomov TE (2012). Disentangling subpopulations in single-molecule FRET and ALEX experiments with photon distribution analysis. Biophys. J..

[79] Kudryavtsev V (2012). Combining MFD and PIE for accurate single‐pair Förster resonance energy transfer measurements. ChemPhysChem.

[80] Lee NK (2005). Accurate FRET measurements within single diffusing biomolecules using alternating-laser excitation. Biophys. J..

[81] Bustamante C, Marko JF, Siggia ED, Smith S (1994). Entropic elasticity of lambda-phage DNA. Science.

