# Supplementary material for: High force catch bond mechanism of bacterial adhesion in the human gut
Source: Nat Commun. 2020 Aug 28;11:4321. doi: 10.1038/s41467-020-18063-x (PMC7456326; doi:10.1038/s41467-020-18063-x)
Supplement: Supplementary file 1 — Supplementary Information [file 41467_2020_18063_MOESM1_ESM.pdf]

High Force Catch Bond Mechanism of Bacterial Adhesion in the Human Gut

Liu et al.

Supplementary Information

High Force Catch Bond Mechanism of Bacterial Adhesion in the Human Gut

Zhaowei Liu[1,2], Haipei Liu[1,2], Andrés M. Vera[3], Rafael C. Bernardi[4,5], Philip Tinnefeld[3], and Michael A. Nash[*,1,2]

[1] Institute of Physical Chemistry, Department of Chemistry, University of Basel, 4058 Basel, Switzerland

[2] Department of Biosystems Science and Engineering, ETH Zurich, 4058 Basel, Switzerland

[3] Faculty of Chemistry and Center for NanoScience, Ludwig-Maximilians-Universität München, Munich, Germany

[4] Beckman Institute for Advanced Science and Technology, University of Illinois at Urbana-Champaign, 61801 Urbana, IL, USA

[5] Department of Physics, Auburn University, 36849 Auburn, AL, USA

Table of contents

Supplementary Figures **1**

Supplementary Figure 1. Amino acid sequence and secondary structure elements of dockerin. 1

Supplementary Figure 2. Model of binding interface between dockerin and cohesin in both binding modes. 2

Supplementary Figure 3. Example force-extension curves obtained in AFM-SMFS measurements. 3

Supplementary Figure 4 SMD simulations of Doc:Coh complex lacking the XMod. 4

Supplementary Figure 5. Rupture force *vs.* loading rate plot from *in vitro* and *in silico* measurements. 5

Supplementary Figure 6. Correction of XMod unfolding force taking biasing effect into account. 6

Supplementary Figure 7. XMod-Doc:Coh complex rupture force histograms at different pulling speeds. 7

Supplementary Figure 8. Fitting the force-dependent off rate data with υ=2/3. 8

Supplementary Figure 9. Combined rupture force histograms of pathways 1 and 2 and calculation of force-dependent off rate. 9

Supplementary Figure 10. Rupture force histograms of binding mode mutants. 10

Supplementary Figure 11. AFM measurements with I27 fingerprint domain. 11

Supplementary Figure 12. AFM measurement of AF647 labeled BMA-KO. 12

Supplementary Figure 13. Monte-Carlo simulation results of complex rupture forces. 13

Supplementary Figure 14. Monte-Carlo simulation results on XMod unfolding force. 14

Supplementary Figure 15. Force clamp Monte-Carlo simulation. 15

Supplementary Table 16

Supplementary Table 1. Kinetic parameters extracted from Monte-Carlo simulations 16

Supplementary Notes 17

Supplementary Note 1. Amino acid sequences 17

Supplementary Note 2. Dudko-Hummer-Szabo model describing the loading rate dependency of rupture forces 19

Supplementary Note 3. Quantifying dual-binding mode behavior using fingerprint domain biasing effect 19

Supplementary References 20

Supplementary Figures 1-15

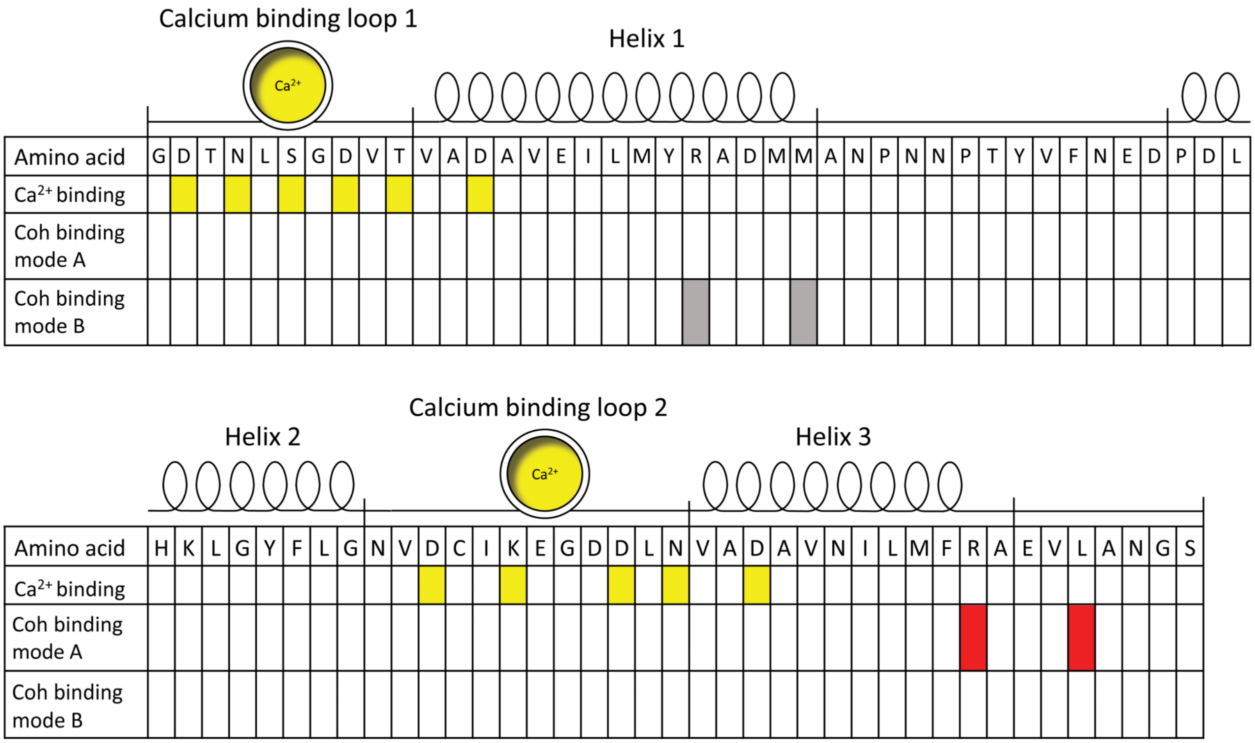

Supplementary Figure 1. Amino acid sequence and secondary structure elements of dockerin. The residues involved in calcium binding are shown in yellow, the residues mutated to knock out the binding mode A are shown in red and the residues mutated to knock out the binding mode B are shown in grey.

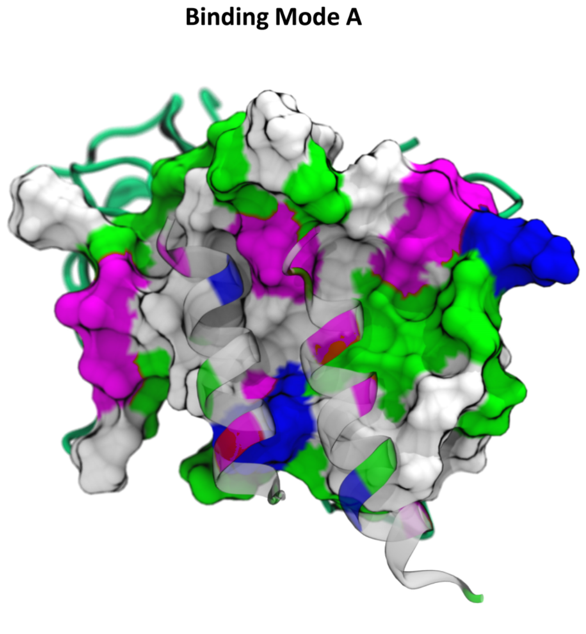

Supplementary Figure 2. Model of binding interface between dockerin and cohesin in both binding modes. In both binding modes, the dockerin and cohesin residues at the binding interface have complementary physical properties, forming a hydrophobic core surrounded by hydrophilic residues. Magenta: negatively charged residues; blue: positive charged residues; white: non-polar residues and green: polar residues.

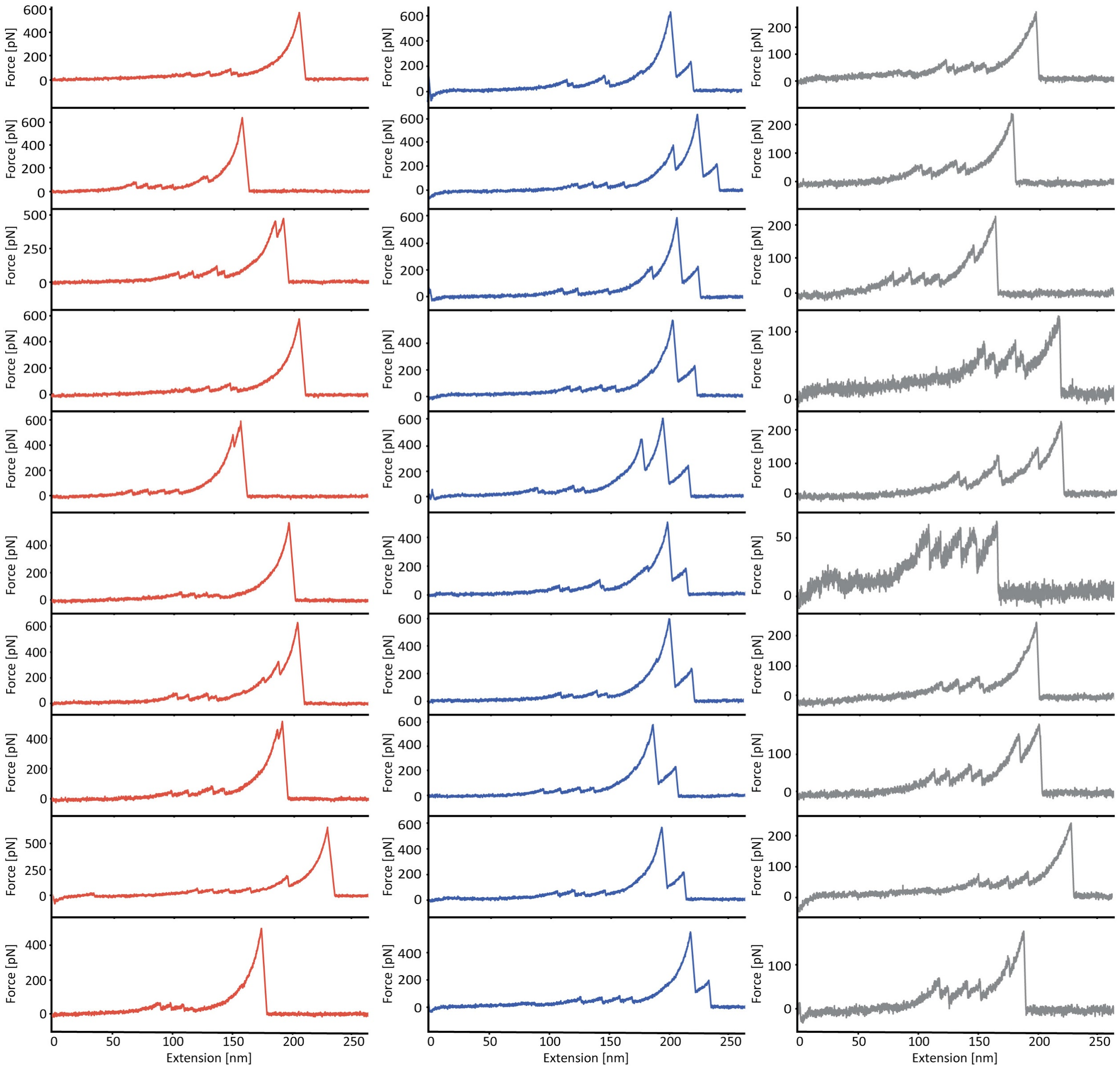

Supplementary Figure 3. Example force-extension curves obtained in AFM-SMFS measurements. Examples of pathways 1 (left column, red), 2 (middle column, blue) and 3 (right column, grey) force-extension curves obtained in AFM-SMFS measurements of WT XMod-Doc:Coh complex. Some force curves showed unassigned unfolding events between ddFLN4 unfolding and complex rupture or XMod unfolding. These unfolding events broadened the contour length histogram and can be attributed to partial unfolding of Coh or Doc domains.

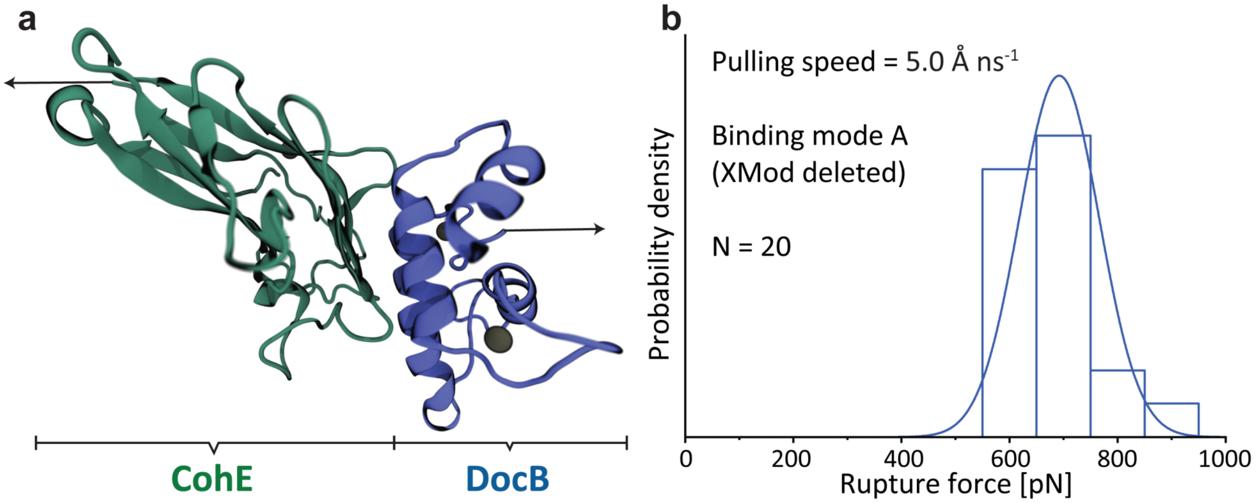

Supplementary Figure 4 SMD simulations of Doc:Coh complex lacking the XMod. a: Structural model showing the Doc:Coh complex in binding mode A. The XMod was deleted from the Doc to mimic the complex in pathway 2. The pulling directions are marked with black arrows. **b:** The rupture force of Doc:Coh complex in binding mode A was measured by performing 20 SMD replicas at 5.0 Å ns^{-1} pulling speed (same as Fig. 1 bc) and plotted in a histogram. The histogram was fitted with a Gaussian distribution, giving the most probable rupture force of 692 pN.

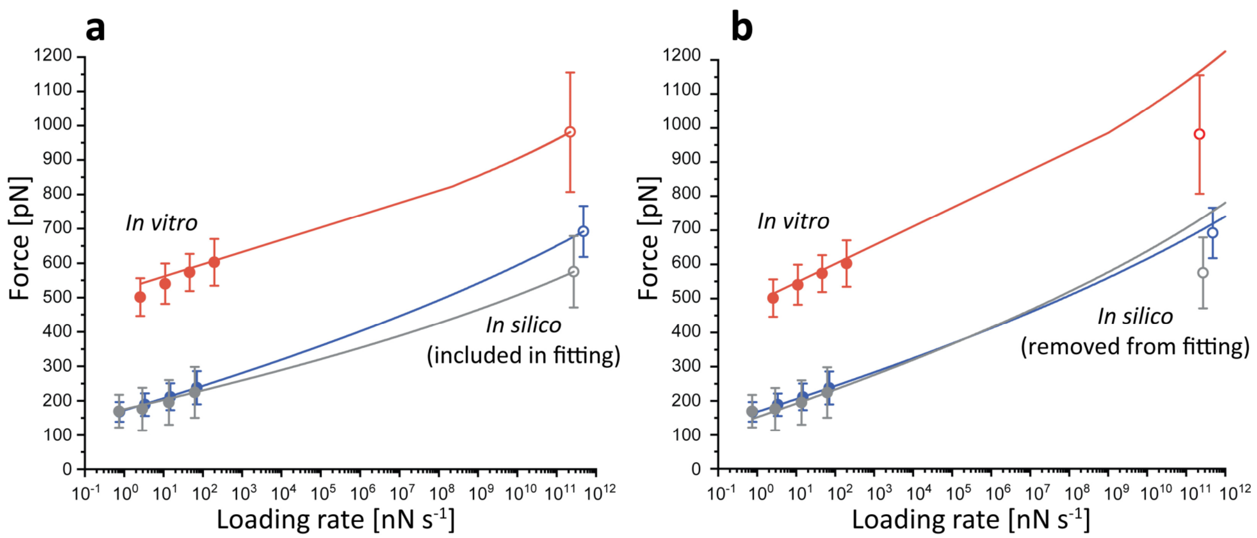

Supplementary Figure 5. Rupture force *vs.* loading rate plot from *in vitro* and *in silico* measurements. The most probable rupture forces measured with AFM-SMFS (*in vitro*) and SMD simulations (*in silico*) were plotted against the loading rate and fitted using the Dudko-Hummer-Szabo model (see Supplementary Note 2) for pathways 1 (red), 2 (blue) and 3 (grey). The SMD simulation results (open circles) were included in the fit **(a)** or excluded from the fit **(b)**. In both cases, the fitted curve showed the same trend and the SMD data was in good agreement with the experimental data. Error bars represent the standard deviation of rupture forces (n=74-317 for *in vitro* measurements, n=200 for *in silico* measurements of pathways 1 and 3 and n=20 for *in silico* measurements of pathway 2).

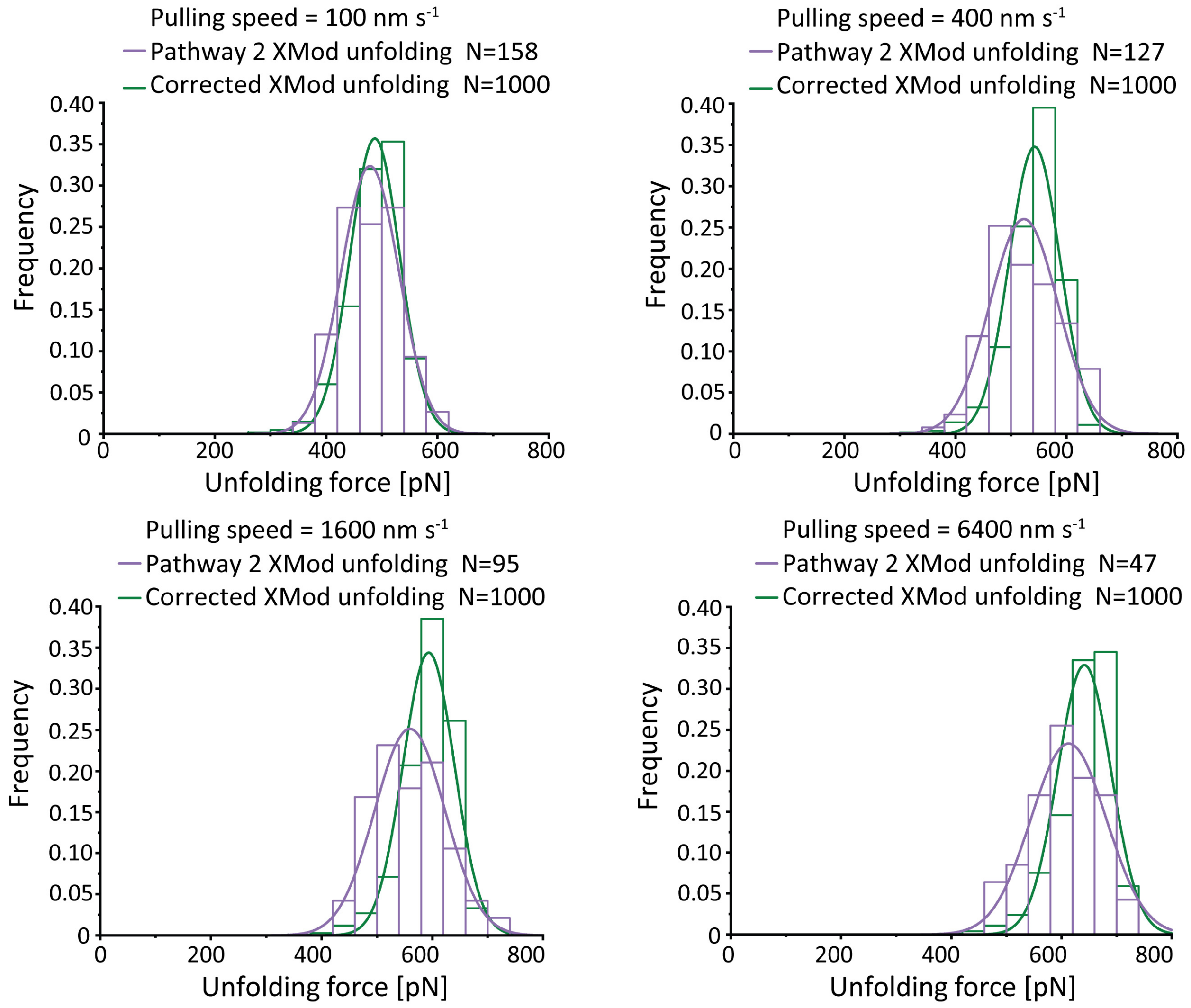

Supplementary Figure 6. Correction of XMod unfolding force taking biasing effect into account. XMod unfolding forces measured in pathway 2 under four different pulling speeds were plotted as histograms and fitted with Gaussian distributions (purple). The XMod unfolding force distributions were corrected using iterative fitting which takes the biasing effect[1] into account (green).

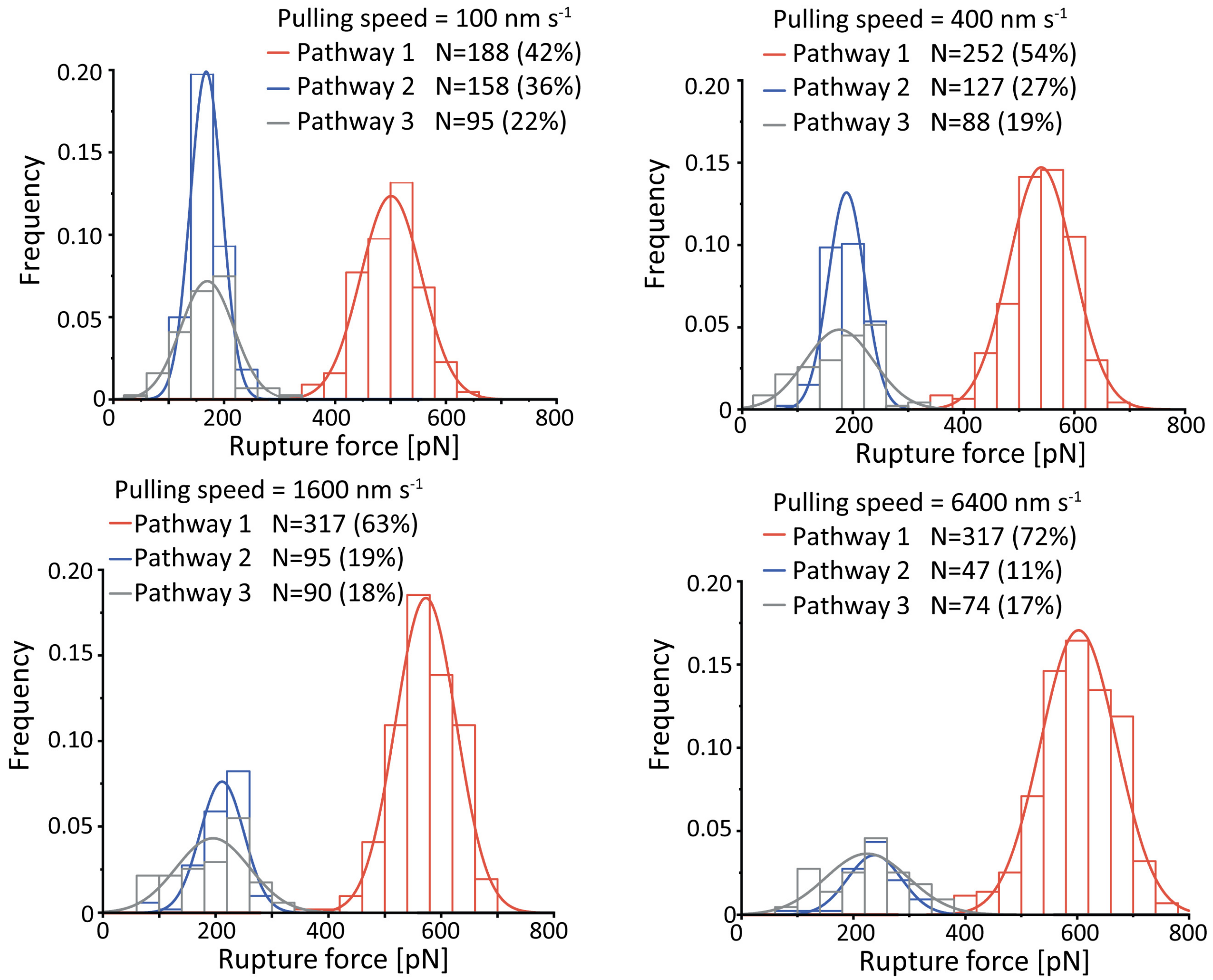

Supplementary Figure 7. XMod-Doc:Coh complex rupture force histograms at different pulling speeds. The three different pathways are plotted in different colors: High rupture force (P1, red); XMod unfolded, low rupture force (P2, blue); and XMod folded, low rupture force (P3, grey). Each histogram was fitted with a Gaussian distribution. Loss of the P2 population at higher pulling speeds drives catch bond behavior in the force ramp scenario.

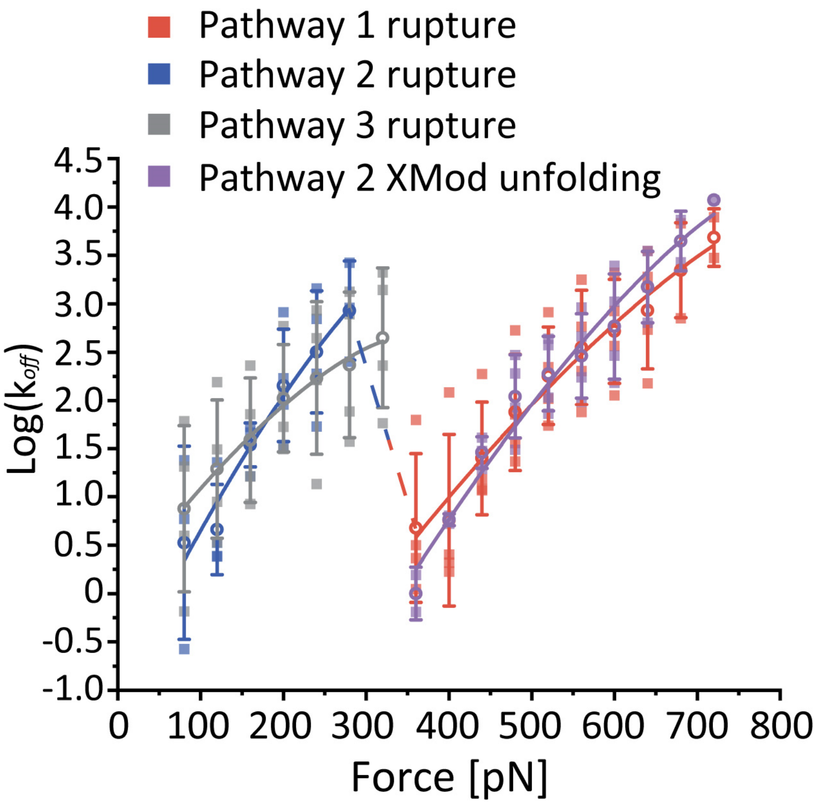

Supplementary Figure 8. Fitting the force-dependent off rate data with υ=2/3. The force-dependent off-rate from Fig. 2f was fitted to the analytical expression (Eq. 5) with υ=2/3 (straight lines). Error bars represent the standard deviation of off-rate (n=2-4). The fitted $\Delta x^{\ddagger}$, $\Delta G^{\ddagger}$ and k_0 values are listed in Table 1.

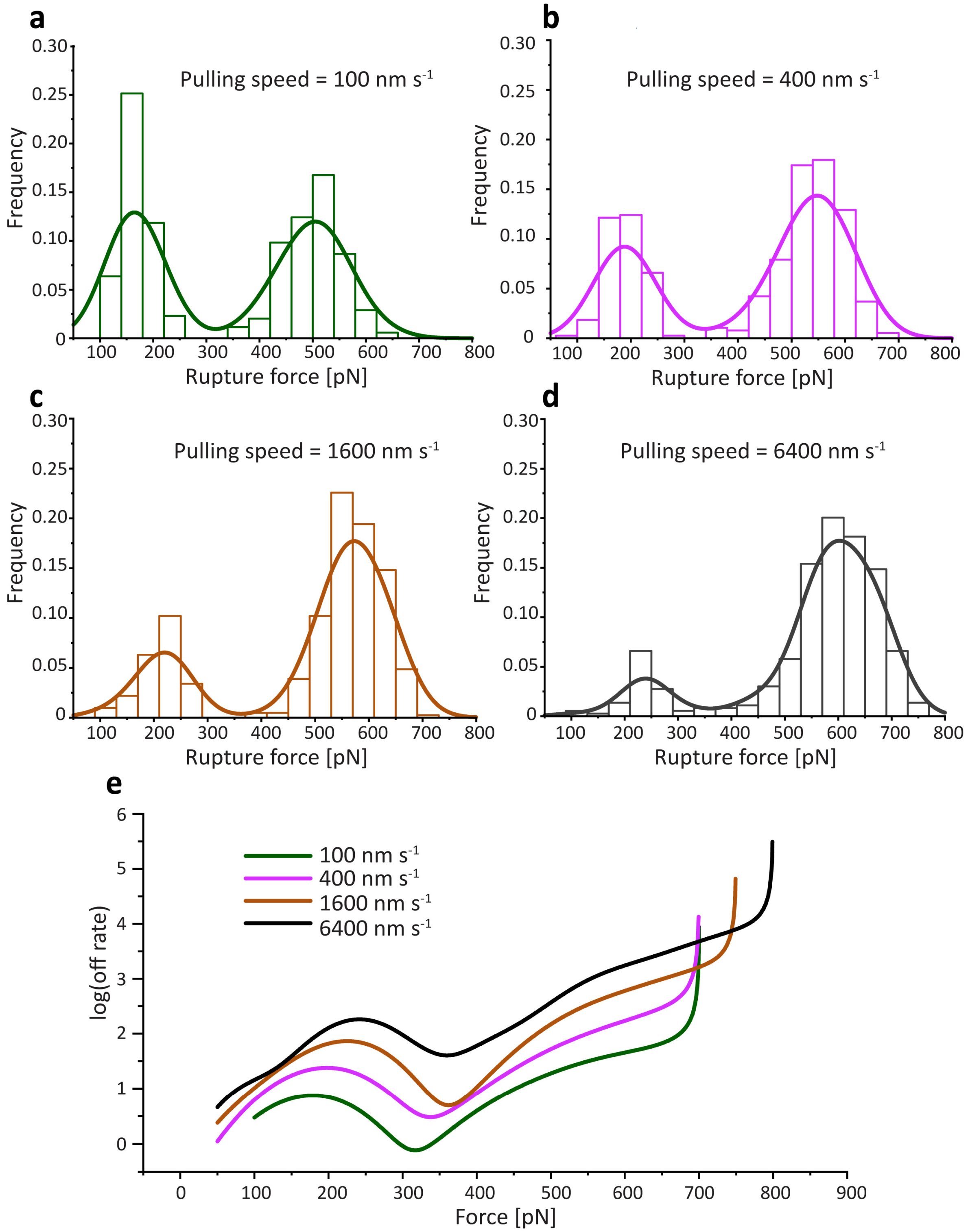

Supplementary Figure 9. Combined rupture force histograms of pathways 1 and 2 and calculation of force-dependent off rate. a-d: Combined rupture force histograms of pathways 1 and 2 at different pulling speeds smoothed with kernel density estimation $P(F)$. **e:** Force-dependent off rate calculated from the kernel density estimation of combined histograms using Eq. 6.

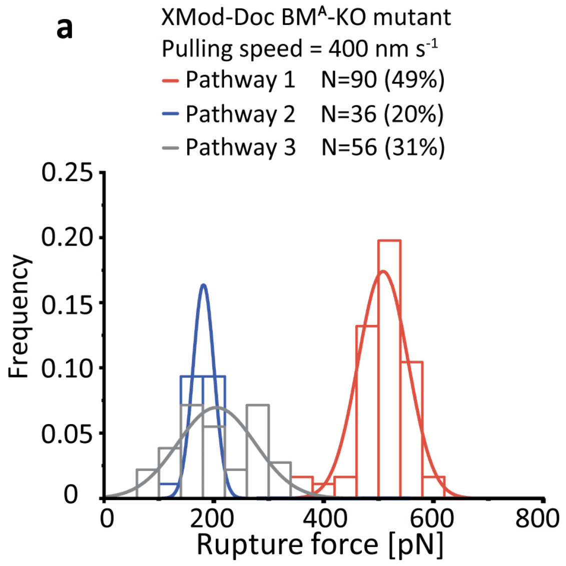

Supplementary Figure 10. Rupture force histograms of binding mode mutants. a: The rupture force histogram measured using BM^A-KO mutant, showing a decreased percentage of pathways 1 and 2 curves. **b:** The rupture force histogram measured using BM^B-KO mutant, showing a decreased percentage (nearly total loss) of pathway 3 curves.

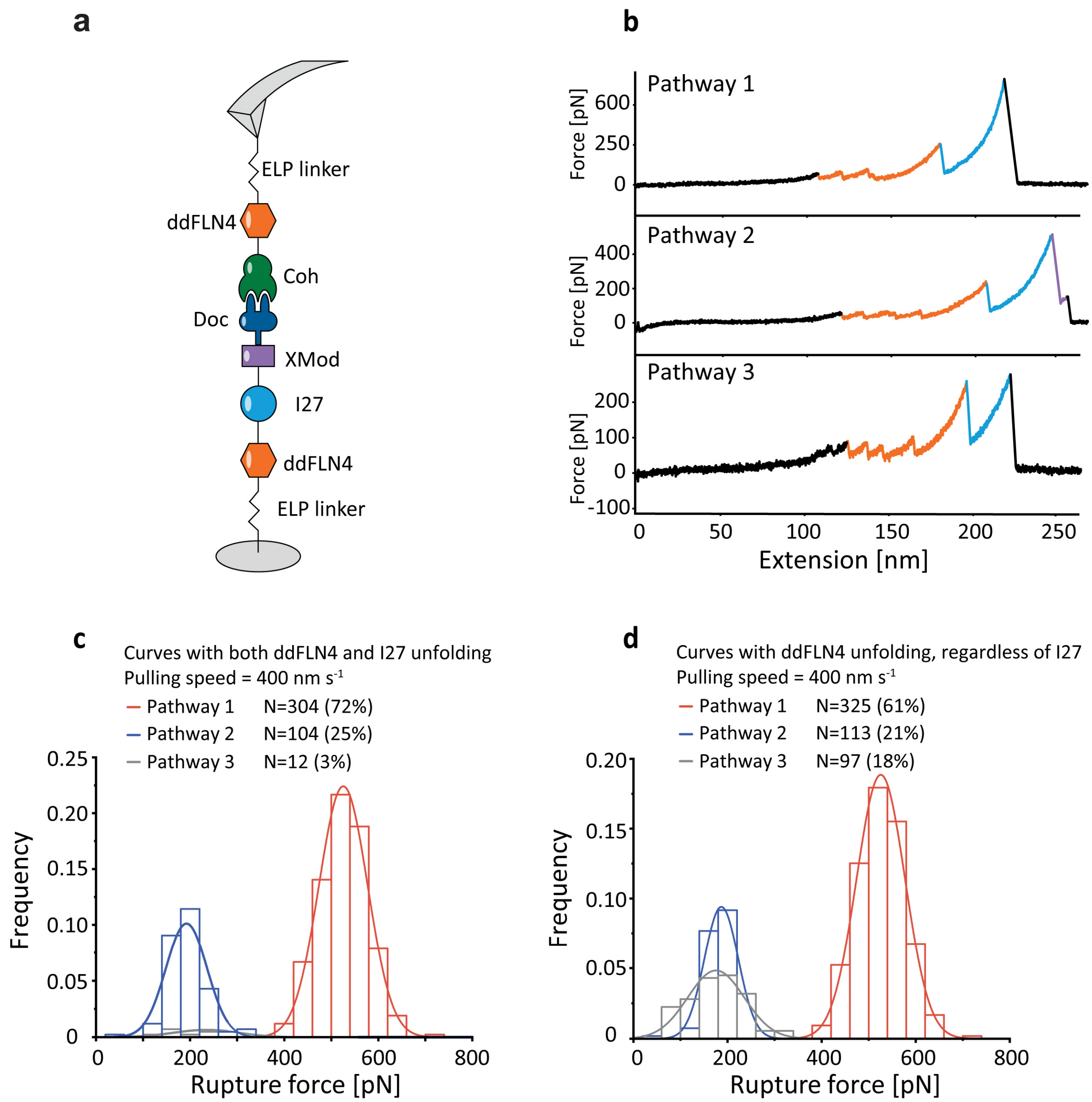

Supplementary Figure 11. AFM measurements with I27 fingerprint domain. a: AFM setup using the I27 biasing effect to demonstrate two binding modes. **b:** Example force-extension curves showing unfolding of 2x ddFLN4 (in orange) and I27 (in blue) in all three pathways. **c:** Rupture force histogram of force curves filtered with both ddFLN4 and I27 fingerprint domains shows that complexes capable of unfolding I27 rarely (3%) dissociated along pathway 3. **d:** Rupture force histogram of force curves filtered with only ddFLN4 showed that pathway 3 was prevalent in the dataset to the same degree as for WT (~18%), but these curves lacked I27 unfolding events.

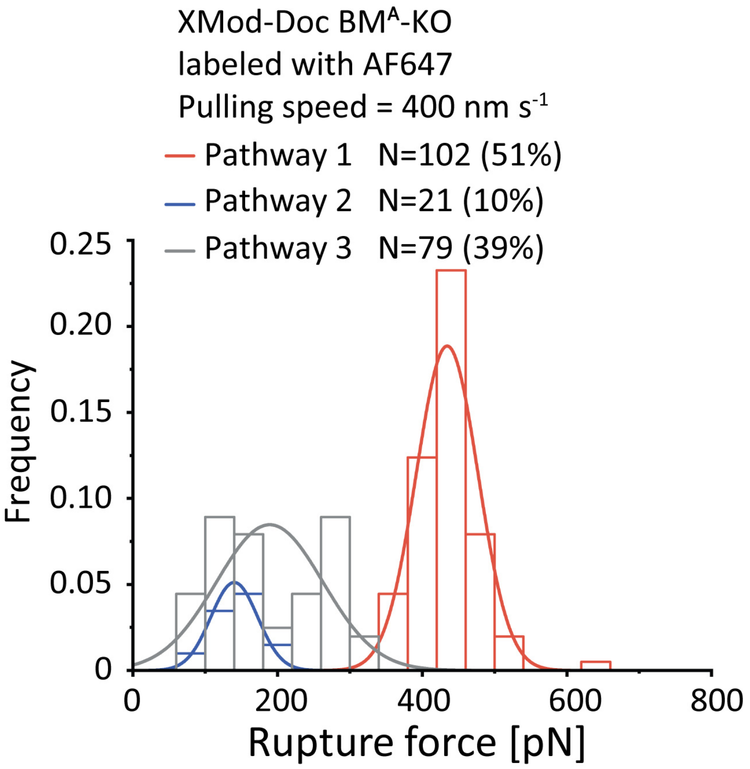

Supplementary Figure 12. AFM measurement of AF647 labeled BM^A-KO. Given the significant molecular weight of the FRET acceptor dye DBCO-AF647 (~1100 grams/mol) and its proximity to the binding interface in binding mode A, we sought to further understand the influence of dye labeling on binding. We used AFM-SMFS to measure the rupture forces between unlabeled wild-type Coh and BM^A-KO labeled with AF647 at 400 nm/s pulling speed. Binding mode A (P1 + P2) consists of 61% of the force curves, which is lower than the unlabeled BM^A-KO mutant (69%), indicating a decrease in the on-rate of the complex in binding mode A. In addition, the most probable rupture force of P1 was found to be 434 pN for the fluorophore labeled BM^A-KO complex, which is significantly lower than the P1 rupture force measured using unlabeled BM^A-KO (Supplementary Fig.10a, 508 pN). The decrease of rupture force in binding mode A for the dye-labeled construct indicated an increase in the intrinsic off-rate of binding mode A as compared with the unlabeled construct. Therefore, we concluded that the AF647 fluorophore at the C-terminus of XMod-Doc slightly destabilized the complex in binding mode A and decreased the binding affinity in this binding mode. However, fluorophore labeling did not have a significant influence on the rupture force of P3, which corresponds to binding mode B. As a consequence, the binding mode A population was less prevalent than binding mode B in the smFRET measurement. The observed ratios of the two binding modes probed by smFRET reflect the equilibrium scenario, whereas binding mode ratios probed by AFM are governed by differences in on-rates only.

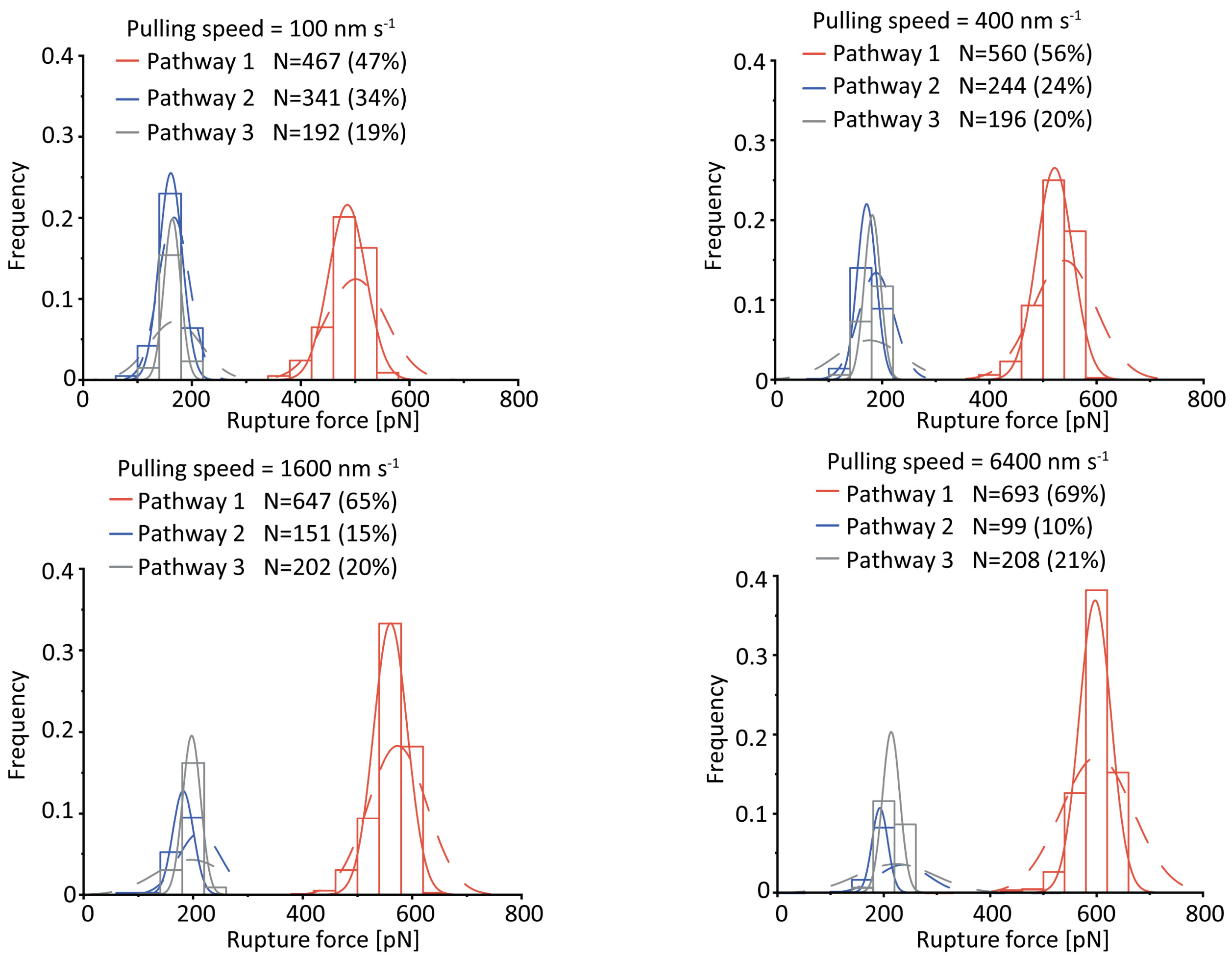

Supplementary Figure 13. Monte-Carlo simulation results of complex rupture forces. The complex rupture forces were simulated at different pulling speeds and plotted as histograms. The histograms were fitted with Gaussian distributions (solid lines). The corresponding experimental rupture force distributions are shown as dashed lines.

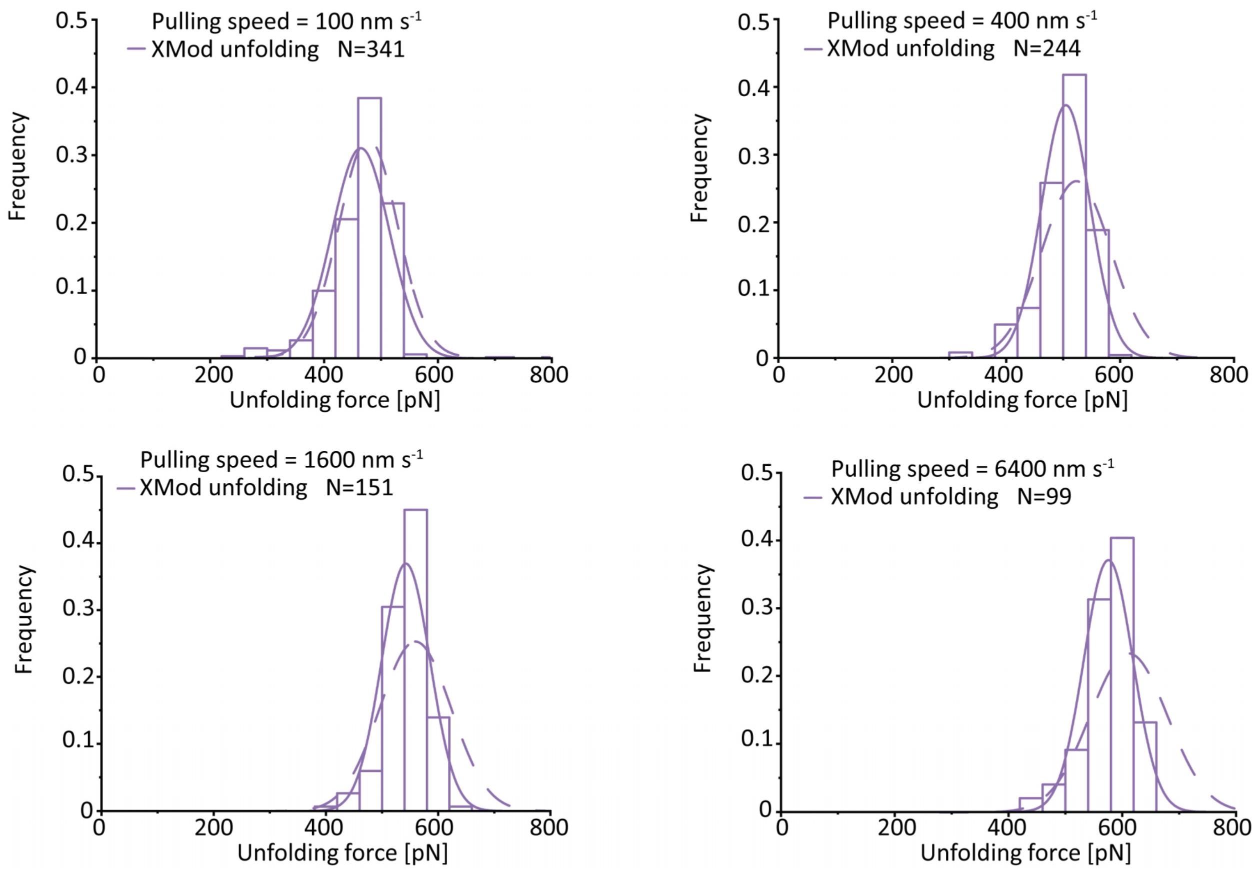

Supplementary Figure 14. Monte-Carlo simulation results on XMod unfolding force. The XMod unfolding forces in pathway 2 were extracted from Monte Carlo simulations at different pulling speeds and plotted into histograms. The histograms were fitted with Gaussian distributions (solid lines). The corresponding experimental unfolding force distribution is shown as a dashed line.

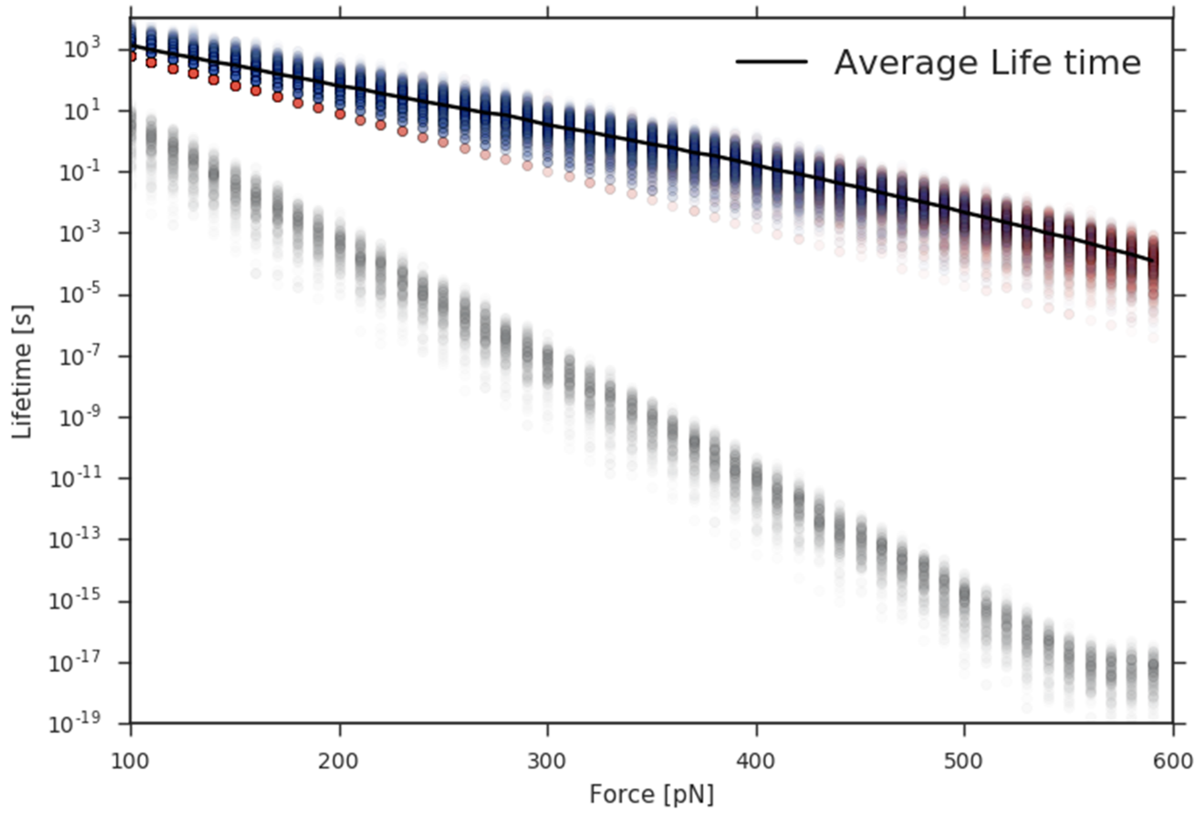

Supplementary Figure 15. Force clamp Monte-Carlo simulation. The force-dependent lifetime of the complex was simulated in force clamp mode (red: pathway 1, blue: pathway 2, grey: pathway 3). The average lifetime (black line) does not exhibit catch bond behavior for the system probed in force clamp mode.

Supplementary Table

Supplementary Table 1. Kinetic parameters extracted from Monte-Carlo simulations

Pathway	Event	k_0 [s^{-1}] (Bell-Evans)	$\Delta x^{\ddagger}$ (Bell-Evans)
1	High force rupture (XMod intact)	1.05×10^{-6}	0.162
2	Unfolding of XMod (measured)	2.81×10^{-6}	0.161
	Low force rupture after XMod unfold	1.34×10^{-4}	0.371
3	Low force rupture (XMod intact)	5.95×10^{-5}	0.366

Supplementary Notes

Supplementary Note 1. Amino acid sequences

Color codes: ybbr-tag, his-tag, ddFLN4, I27, ELP linker, XMod, Doc, Coh, Avi-tag, inter-domain linker, mutations (AzF = p-azido-phenylalanine).

Addgene #153439: pET28a-ybbr-ELP-ddFLN4-XMod-Doc-HIS (wild type)

GTDSLEFIASKLAWGSGHGVGVPGMGVPGVGVPGVGVPGVGVPGVGVPGVGVPGVGV
PGVGVPGEGVPGEGVPGVGVPGMGVPGVGVPGVGVPGVGVPGVGVPGVGVPGVGVP
GVGVPGEGVPGEGVPGVGVPGMGVPGVGVPGVGVPGVGVPGVGVPGVGVPGVGVPG
VGVPGEGVPGEGVPGWPSGSADPEKSYAEGPGLDGGECFQPSKFKIHAVDPDGVHRTD
GGDGFVVTIEGPAPVDPVMVDNGDGTYDVEFEPKEAGDYVINLTLDGDNVNGFPKTVT
VKPAPGSGSGSGSSGSETTTETSVSQSFSFTEPTRVCYWSHDNRPFDLEGLKVILQETIFII
HEDGTVVEAEEQPAAVDITEQCMTIFCNDKQVATPEDTYETQIYKYMLEFAYEGNPDVI
VGTHQVLIGVKGDTNLSGDVTVADAVEILMYRADMMANPNNPTYVFNEDPDLHKLGY
FLGNVDCIKEGDDLNVADAVNILMFRAEVLANGSATWEQIVGYDLIDPSDESTFRTIEKL
GSHHHHHH

Addgene #153440 pET28a-ybbr-ELP-ddFLN4-XMod-Doc-HIS (BMA-KO)

GTDSLEFIASKLAWGSGHGVGVPGMGVPGVGVPGVGVPGVGVPGVGVPGVGVPGVGV
PGVGVPGEGVPGEGVPGVGVPGMGVPGVGVPGVGVPGVGVPGVGVPGVGVPGVGVP
GVGVPGEGVPGEGVPGVGVPGMGVPGVGVPGVGVPGVGVPGVGVPGVGVPGVGVPG
VGVPGEGVPGEGVPGWPSGSADPEKSYAEGPGLDGGECFQPSKFKIHAVDPDGVHRTD
GGDGFVVTIEGPAPVDPVMVDNGDGTYDVEFEPKEAGDYVINLTLDGDNVNGFPKTVT
VKPAPGSGSGSGSSGSETTTETSVSQSFSFTEPTRVCYWSHDNRPFDLEGLKVILQETIFII
HEDGTVVEAEEQPAAVDITEQCMTIFCNDKQVATPEDTYETQIYKYMLEFAYEGNPDVI
VGTHQVLIGVKGDTNLSGDVTVADAVEILMYRADMMANPNNPTYVFNEDPDLHKLGY
FLGNVDCIKEGDDLNVADAVNILMFAAEVEANGSATWEQIVGYDLIDPSDESTFRTIEKL
LGSHHHHHH

Addgene #153441 pET28a-ybbr-ELP-ddFLN4-XMod-Doc-HIS (BM^{B}-KO)

GTDSLEFIASKLAWGSGHGVGVPGMGVPGVGVPGVGVPGVGVPGVGVPGVGVPGVGV
PGVGVPGEGVPGEGVPGVGVPGMGVPGVGVPGVGVPGVGVPGVGVPGVGVPGVGVP
GVGVPGEGVPGEGVPGVGVPGMGVPGVGVPGVGVPGVGVPGVGVPGVGVPGVGVPG
VGVPGEGVPGEGVPGWPSGSADPEKSYAEGPGLDGGECFQPSKFKIHAVDPDGVHRTD
GGDGFVVTIEGPAPVDPVMVDNGDGTYDVEFEPKEAGDYVINLTLDGDNVNGFPKTVT
VKPAPGSGSGSGSSGSETTTETSVSQSFSFTEPTRVCYWSHDNRPFDLEGLKVILQETIFII
HEDGTVVEAEEQPAAVDITEQCMTIFCNDKQVATPEDTYETQIYKYMLEFAYEGNPDVI
VGTHQVLIGVKGDTNLSGDVTVADAVEILMYAADMEANPNNPTYVFNEDPDLHKLGY

FLGNVDCIKEGDDLNVADAVNILMFRAEVLANGSATWEQIVGYDLIDPSDESTFRTIEKL
GSHHHHHH

Addgene #153442 pET28a-ybbr-HIS -ELP-ddFLN4-I27-XMod-Doc (wild type)

GTDSLEFIASKLAHHHHHHWGSGHGVGVPGMGVPGVGVPGVGVPGVGVPGVGVPGV
GVPGVGVPGVGVPGEGVPGEGVPGVGVPGMGVPGVGVPGVGVPGVGVPGVGVPGVG
VPGVGVPGVGVPGEGVPGEGVPGVGVPGMGVPGVGVPGVGVPGVGVPGVGVPGVGV
PGVGVPGVGVPGEGVPGEGVPGWPSGSADPEKSYAEGPGLDGGECFQPSKFKIHAVDP
DGVHRTDGGDGFVVTIEGPAPVDPVMVDNGDGTYDVEFEPKEAGDYVINLTLDGDNV
NGFPKTVTVKPAPLIEVEKPLYGVEVFVGETAHFEIELSEPDVHGQWKLKGQPLAASPD
CEIIEDGKKHILILHNCQLGMTGEVSFQAANTKSAANLKVKELGSGSGSGSSGSETTTETS
VSQSFSFTEPTRVCYWSHDNRPFDLEGLKVILQETIFIIHEDGTVVEAEEQPAAVDITEQC
MTIFCNDKQVATPEDTYETQIYKYMLEFAYEGNPDVIVGTHQVLIGVKGDTNLSGDVTV
ADAVEILMYRADMMANPNNPTYVFNEDPDLHKLGYFLGNVDCIKEGDDLNVADAVNI
LMFRAEVLANGSATWEQIVGYDLIDPSDESTFRTIEKL

Addgene #153443 pET28a-ybbr-XMod-Doc (WT, S199AzF)-HIS

GTDSLEFIASKLAGSSGSETTTETSVSQSFSFTEPTRVCYWSHDNRPFDLEGLKVILQETIF
IIHEDGTVVEAEEQPAAVDITEQCMTIFCNDKQVATPEDTYETQIYKYMLEFAYEGNPDV
IVGTHQVLIGVKGDTNLSGDVTVADAVEILMYRADMMANPNNPTYVFNEDPDLHKLG
YFLGNVDCIKEGDDLNVADAVNILMFRAEVLANGAzFATWEQIVGYDLIDPSDESTFRTI
EKLGSHHHHHH

Addgene #153444 pET28a-ybbr-XMod-Doc (BM^{A}-KO, S199AzF)-HIS

GTDSLEFIASKLAGSSGSETTTETSVSQSFSFTEPTRVCYWSHDNRPFDLEGLKVILQETIF
IIHEDGTVVEAEEQPAAVDITEQCMTIFCNDKQVATPEDTYETQIYKYMLEFAYEGNPDV
IVGTHQVLIGVKGDTNLSGDVTVADAVEILMYRADMMANPNNPTYVFNEDPDLHKLG
YFLGNVDCIKEGDDLNVADAVNILMFAAEVEANGAzFATWEQIVGYDLIDPSDESTFRTI
EKLLGSHHHHHH

Addgene #153445 pET28a-ybbr-XMod-Doc (BM^{B}-KO, S199AzF)-HIS

GTDSLEFIASKLAGSSGSETTTETSVSQSFSFTEPTRVCYWSHDNRPFDLEGLKVILQETIF
IIHEDGTVVEAEEQPAAVDITEQCMTIFCNDKQVATPEDTYETQIYKYMLEFAYEGNPDV
IVGTHQVLIGVKGDTNLSGDVTVADAVEILMYRADMMANPNNPTYVFNEDPDLHKLG
YFLGNVDCIKEGDDLNVADAVNILMFRAEVLANGAzFATWEQIVGYDLIDPSDESTFRTI
EKLGSHHHHHH

Addgene #153446 pET28a-Coh-ddFLN4-ELP-HIS-ybbr

ADGAAKLSMDQKFAEPGETVEIALNLENFDASWTGLEFLVNYDPKLEVALDGAGDIDYSYGDAIGAMGKKISVGGAISKDLTADGLKGFAFAWGTATAISGNGQLGVFKFTVPADAQPGDEFPVNLTVNVGSFIDANKENIPFETVNGWIKIKEEGSGSGSGSADPEKSYAEGPGLDGGECFQPSKFKIHAVDPDGVHRTDGGDGFVVTIEGPAPVDPVMVDNGDGTYDVEFEPKEAGDYVINLTLDGDNVNGFPKTVTVKPAPGSGSGSHGVGVPGMGVPGVGVPGVGVPGVGVPGVGVPGVGVPGVGVPGVGVPGEGVPGEGVPGVGVPGMGVPGVGVPGVGVPGVGVPGVGVPGVGVPGVGVPGVGVPGVGVPGEGVPGEGVPGVGVPGMGVPGVGVPGVGVPGVGVPGVGVPGVGVPGVGVPGVGVPGVGVPGEGVPGEGVPGWRGHHHHHHGSDSLEFIASKLA

Addgene #153447 pET28a-Avi-Coh (E154C)-HIS

GLNDIFEAQKIEWHEGSGSADGAAKLSMDQKFAEPGETVEIALNLENFDASWTGLEFLVNYDPKLEVALDGAGDIDYSYGDAIGAMGKKISVGGAISKDLTADGLKGFAFAWGTATAISGNGQLGVFKFTVPADAQPGDEFPVNLTVNVGSFIDANKENIPFETVNGWIKIKCEGSHHHHHH

Supplementary Note 2. Dudko-Hummer-Szabo model describing the loading rate dependency of rupture forces

The rupture force-loading rate plots in Supplementary Fig. 5 were fitted using the following equation[2]:

$$F = \frac{\Delta G^{\ddagger}}{\nu \Delta x^{\ddagger}}[1 - (\frac{1}{\Delta G^{\ddagger}} \ln \frac{k_0 e^{-\beta \Delta G^{\ddagger}}}{\Delta x^{\ddagger} l})^{\nu}]$$

where k_0 is the intrinsic off rate in the absence of force, $\Delta x^{\ddagger}$ is the distance to the energy barrier, $\Delta G^{\ddagger}$ is the height of the energy barrier in the absence of force, $\beta^{-1}=k_B T$, and $\upsilon = 0.5$, assuming the shape of the free-energy surface is cusp.

Supplementary Note 3. Quantifying dual-binding mode behavior using fingerprint domain biasing effect

As shown in Supplementary Fig. 11a, a titin I27 domain, which under our conditions has an unfolding force around ~200 pN[3,4], was inserted between ddFLN4 and WT XMod-Doc as an additional fingerprint domain. The interaction between XMod-Doc and Coh was then probed using AFM-SMFS in the presence of two ddFLN4 and one I27 fingerprint domain. Unfolding of I27 was identified by its unfolding force of ~200 pN and the contour length increment of ~28 nm, as shown in Supplementary Fig. 11b. Force-extension curves were screened based on the contour length

increments given by two ddFLN4 domains and one I27 domain and then sorted into the aforementioned three pathways based on the rupture force of the complex and the folding state of XMod. The rupture force of each pathway was plotted in a rupture force histogram (Supplementary Fig. 11c). In binding mode A, the complex was able to resist an external force up to ~500 pN prior to rupture, which was larger than the force required to unfold I27. Therefore, pathways 1 and 2 were not biased by the additional I27 domain and were still observable in the dataset. However, binding mode B has relatively low mechanical stability and ruptures prior to I27 unfolding. Therefore, the frequency of pathway 3 was significantly decreased to only 3% when using I27 as an additional fingerprint domain for curve selection. However, when screening the force curves only based on the two ddFLN4 domains regardless of whether the curves contained I27 unfolding or not, the frequency of different pathways in the screened curves (Supplementary Fig. 11d) was the same as the construct lacking I27 (Supplementary Fig. 6b). This observation further demonstrated that pathways 1 and 2 (high force curves) belong to a different discrete binding mode than pathway 3 (low force curves). The two binding modes have different mechanical stabilities and cannot be converted to the other one on the timescale of the AFM-SMFS curve (~1 second).

Supplementary References

1. Schoeler, C., Verdorfer, T., Gaub, H. E. & Nash, M. A. Biasing effects of receptor-ligand complexes on protein-unfolding statistics. *Phys Rev E* **94**, 042412 (2016).

2. Dudko, O. K., Hummer, G. & Szabo, A. Intrinsic rates and activation free energies from single-molecule pulling experiments. *Phys. Rev. Lett.* **96**, 108101 (2006).

3. Rief, M., Gautel, M., Oesterhelt, F., Fernandez, J. M. & Gaub, H. E. Reversible unfolding of individual titin immunoglobulin domains by AFM. *Science* **276**, 1109–1112 (1997).

4. Liu, H., Ta, D. T. & Nash, M. A. Mechanical Polyprotein Assembly Using Sfp and Sortase-Mediated Domain Oligomerization for Single-Molecule Studies. *Small Methods* **2**, 1800039 (2018).
